# A Review of Optical Metrology Techniques for Advanced Manufacturing Applications

**DOI:** 10.3390/mi16111224

**Published:** 2025-10-28

**Authors:** Fangyuan Zhao, Hanyao Tang, Xuerong Zou, Xinghui Li

**Affiliations:** Tsinghua Shenzhen International Graduate School, Tsinghua University, Shenzhen 518055, China; fy-zhao24@mails.tsinghua.edu.cn (F.Z.); tang-hy24@mails.tsinghua.edu.cn (H.T.); zouxr24@mails.tsinghua.edu.cn (X.Z.)

**Keywords:** optical metrology, precision manufacturing, positioning technology, surface metrology

## Abstract

Advanced manufacturing places stringent demands on measurement technologies, requiring ultra-high precision, non-contact operation, high throughput, and real-time adaptability. Optical metrology, with its distinct advantages, has become a key enabler in this context. This paper reviews optical metrology techniques from the perspective of precision manufacturing applications, emphasizing precision positioning and surface topography measurement while noting the limitations of traditional contact-based methods. For positioning, interferometers, optical encoders, and time-of-flight methods enable accurate linear and angular measurements. For surface characterization, techniques such as interferometry, structured light profilometry, and confocal microscopy provide reliable evaluation across scales, from large structures to micro- and nano-scale features. By integrating these approaches, optical metrology is shown to play a central role in bridging macroscopic and nano-scale characterization, supporting both structural assessment and process optimization. This review highlights its essential contribution to advanced manufacturing, and offers a concise reference for future progress in high-precision and intelligent production.

## 1. Introduction

Advanced manufacturing serves as the core driving force behind the new wave of technological revolution and industrial transformation, with its developmental level directly impacting the strategic landscape of national competitiveness and comprehensive national strength. In recent decades, nations have invested in advanced manufacturing research to enhance their competitive edge in global markets, driven by process efficiency, precision, flexibility, and sustainability. For instance, the United States President’s Council of Advisors on Science and Technology (PCAST) proposed the national-level ‘Advanced Manufacturing Partnership (AMP)’ strategic vision in its 2012 report, aiming to identify emerging technologies to maintain US manufacturing leadership [1]; meanwhile, the European Union established digitalisation, greening, and flexible manufacturing as the core pillars of advanced manufacturing in its ‘Factories of the Future’ strategic roadmap, emphasising the critical importance of advanced manufacturing processes as one of five key areas for EU industrial competitiveness and sustainability [2]. As a foundational enabler for the transformation and upgrading of traditional industries and the cultivation of emerging future industries, advanced manufacturing has become a strategic battleground for major industrial nations vying for dominance in future economic development.

Advanced manufacturing technology is not a singular technical breakthrough, but rather a complex ecosystem encompassing advanced equipment, cutting-edge components, innovative processes, and intelligent management. Its core manifests in four key aspects: firstly, high-performance advanced manufacturing equipment such as high-precision CNC machine tools, additive manufacturing apparatus, and industrial robots; secondly, critical components with extreme service performance, including aero-engine blades, lithography objectives for integrated circuits, and micro-electro-mechanical system (MEMS) sensors; thirdly, novel manufacturing processes driving paradigm shifts, such as laser processing, ultra-precision polishing, and composite material placement; and fourthly, intelligent manufacturing systems enabling holistic optimisation, including digital twins, flexible production lines, and full lifecycle management. These elements collectively form the grand panorama of modern advanced manufacturing.

At every stage of the advanced manufacturing system, measurement technology plays a pivotal role, serving as the critical link connecting design, processing, and quality control. Whether it concerns the nanometre-level precision positioning of moving equipment components, the micro- and nano-scale measurement of part geometries and surface defects, or the one hundred per cent real-time online inspection and feedback control of processing quality on production lines, measurement technology permeates the entire process of design verification, production monitoring, and quality assessment. Without precise measurement, accurate manufacturing becomes unattainable, and process optimisation or system intelligence remains out of reach. Consequently, the level of measurement technology fundamentally determines the upper limits of precision and efficiency achievable in advanced manufacturing. As modern manufacturing evolves towards higher precision, automation, and intelligence, demands have not only intensified for measurement accuracy (sub-micrometre and even sub-nanometre scales) [3,4,5,6,7,8], but also for non-contact, high-throughput measurement capabilities [9]. Moreover, online/real-time measurement technologies enable feedback on critical process parameters during machining for the timely adjustment of process conditions, further underscoring the vital role of precision measurement in advanced manufacturing.

**Figure 1:**
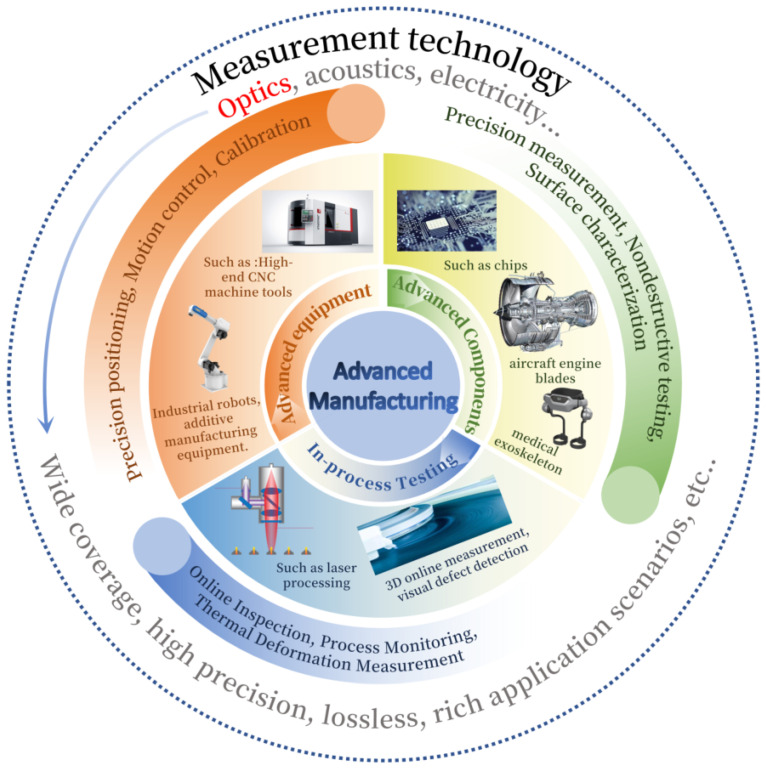
Typical components used in advanced manufacturing, and critical aspects of related measurement technologies.

To meet these demanding measurement requirements, various measurement methods based on different physical principles have emerged, primarily categorised as optical, acoustic, electrical, and traditional contact-based techniques. Among these, optical measurement technology stands out due to its unique advantages: non-contact operation, high precision, high speed, and the ability to capture full-field information. It causes no damage or scratches to delicate or soft workpiece surfaces, acquires three-dimensional topography and contour information at rates of millions of data points per second, and integrates readily with machine vision and automated systems for real-time online monitoring. These characteristics make it perfectly aligned with the core requirements of advanced manufacturing for non-destructive, high-efficiency, precision, and online capabilities, establishing it as a key enabling technology and a frontier research focus driving the advancement of advanced manufacturing. In recent years, advancements in emerging technologies such as optical frequency combs, interference fringe tracking, and digital holographic measurement have significantly enhanced the measurement range, interference resistance, and system integration capabilities of optical measurement techniques [10,11,12,13]. The grand vision of advanced manufacturing, its profound dependence on measurement technologies, and the pivotal role played by optical measurement collectively form a complex and interrelated technological ecosystem. To clearly and systematically elucidate this multi-layered, networked support relationship, this paper constructs an ‘Advanced Manufacturing System and Its Technical Support Spectrum’, depicted in Figure 1.

From the perspective of measurement principles, optical measurement can be categorised into interferometry, optical imaging, spectroscopic measurement, and other hybrid techniques and advanced methods. Optical interferometry has become the cornerstone of high-precision length measurement, delivering unparalleled accuracy across diverse scientific and industrial applications while offering advantages such as high resolution, low cost, and robustness under various environmental conditions [14,15]. Grating encoders feature simple structures and high reliability, with multi-degree-of-freedom grating encoders providing high-precision displacement and angular measurement solutions for various cutting-edge industrial applications [16,17,18]. Chromatography confocal technology is widely applied in profile measurement, biomedical imaging, and thickness measurement [19]. Diffractive large-area grating arrays play a crucial role in long-distance positioning technology [20,21]. Based on their applications within manufacturing, optical measurement technologies broadly serve two primary purposes: techniques for precision positioning, and those for surface-form metrology.

Optical measurement technologies for precision positioning constitute one of the indispensable core technologies used in advanced manufacturing processes. Within advanced manufacturing systems, sub-micrometre or even nanometre-level positioning accuracy forms the foundation for numerous critical stages [22,23]. For instance, in lithography processes, positional deviations between the mask and wafer directly impact pattern transfer precision, thereby determining chip performance [24,25]. In fields such as coordinate measuring and CNC machining, high-precision linear displacement measurement systems based on laser interferometers, optical linear encoders, confocal sensing, and other technologies can effectively enhance machining quality and repeatability [26,27]. Whether considering the impact of Abbe error on the uncertainty of simple linear displacement measurement or the demand for object positioning in three-dimensional space, researchers have increasingly focused on the measurement of out-of-axis angular displacement [3,28,29,30]. Optical angle sensors, primarily based on autocollimators, offer diverse high-precision, low-cost solutions for angular displacement measurement, further advancing multi-degree-of-freedom measurement development and its application in precision manufacturing [31,32].

Surface metrology techniques also serve as critical tools for ensuring product precision and performance within advanced manufacturing systems, playing a particularly vital role in high-end optical processing, semiconductor manufacturing, and aerospace component development. Interferometry, with its high resolution and repeatability, is extensively employed for high-precision freeform and aspheric surface inspection. Using techniques such as computer-generated holography (CGH) or subaperture stitching, interferometric systems can achieve subwavelength-level error analysis on large, complex surfaces [33,34]. Concurrently, structured light measurement technology, characterised by its non-contact and rapid imaging capabilities, has emerged as another vital method for complex topography inspection. Light-field modulation techniques such as digital light processing (DLP), micro-electro-mechanical systems (MEMSs), and diffractive optical elements (DOEs) provide flexible support for diverse measurement tasks. DLP systems are widely employed in industrial metrology due to their high pattern accuracy, while MEMS solutions suit portable and integrated devices [35,36,37]. For micro–nano measurements, interferometric microscopy, white-light interferometry, and confocal microscopy enable nano-scale surface profiling and focal depth assessment. Twyman–Green interferometers are frequently employed for topographical error detection in microlens arrays; conversely, scanning white-light interferometry and laser scanning confocal microscopy combine high resolution with extensive scanning ranges, demonstrating exceptional performance in micro–nano structure measurement [34,38]. In recent years, optical surface metrology has progressively advanced towards higher precision, automation, and integration, driving breakthroughs in the application of complex freeform surfaces within precision manufacturing.

While numerous reviews addressing optical metrology exist, most focus on specific technical categories or solely examine measurement techniques themselves, lacking comprehensive exposition and systematisation from an advanced manufacturing application perspective. To address this gap, this paper synthesises and analyses optical metrology methods employed in contemporary manufacturing for precision positioning and surface quality assessment. Section 2 and Section 3 concentrate on practical precision manufacturing scenarios, detailing the principles and characteristics of mainstream optical metrology solutions across different contexts. Section 2 covers optical techniques for precision positioning, including measurements of linear and angular displacement, alongside positioning systems employed in multi-degree-of-freedom measurements. Section 3 outlines the fundamental principles and latest developments in surface topography metrology methods, encompassing both macro-level surface parameter evaluation and micro-level critical dimension measurement.

## 2. Positioning Technology in Precision Manufacturing

In the field of advanced manufacturing, positioning technology is one of the core enablers for ensuring manufacturing quality and production efficiency. Its performance directly determines machining accuracy, the operational stability of equipment, and product consistency. In precision manufacturing, position sensors used for accurate positioning not only provide real-time location information, but also supply critical data for motion control, closed-loop feedback, and process quality monitoring. For complex machining tasks, high-precision positioning over multiple axes or even in full spatial degrees of freedom is indispensable. Linear displacement characterizes the translational motion of a target, whereas angular displacement describes changes in the target’s spatial orientation. These two types of displacement are often coupled. For example, in multi-degree-of-freedom motion platforms, precision positioning mechanisms, or robotic joints, even minute angular errors can induce significant linear displacement errors, thereby affecting machining accuracy and measurement stability.

Among the various metrology and positioning approaches, optical measurement technologies have been gaining increasing importance in advanced manufacturing due to their advantages of high accuracy, non-contact operation, wide measurement range, rapid response, and ease of integration with automation systems. Compared with contact-based and other non-contact methods, optical techniques typically offer superior resolution, measurement stability, and environmental adaptability, and can address multi-scale measurement requirements ranging from the nanometre to the meter level. With the continuous advances in light sources, detectors, and signal processing techniques, the application boundaries of optical positioning methods are being steadily extended to accommodate increasingly demanding and diverse manufacturing scenarios.

This section classifies the optical positioning methods that are widely used in advanced manufacturing according to their application requirements and operating principles, and presents a review of the latest research progress and emerging trends in this area.

### 2.1. Linear Displacement Measurement

As one of the most fundamental geometric quantities, linear displacement has the widest range of application demands in advanced manufacturing. Whether in semiconductor lithography, precision machine tool spindle and worktable positioning, coordinate measuring machines, additive manufacturing, or high-end assembly, high-accuracy linear displacement measurement is a prerequisite for achieving nanometer- and even sub-nanometer-level control. To meet the evolving requirements driven by the trend toward higher accuracy, longer travel ranges, and superior dynamic performance in manufacturing equipment, linear displacement measurement techniques must not only deliver extremely high resolution and stability, but also ensure high measurement speed, strong environmental adaptability, and ease of system integration.

Table 1 provides a performance and application scenario comparison of the various optical linear displacement measurement techniques used in advanced manufacturing. In this section, these techniques are reviewed from the perspective of their underlying operating principles.

#### 2.1.1. Laser Interferometer

Laser interferometers utilize the wavelength of light as a reference for measurement scales, enabling nano-scale and even sub-nano-scale measurement accuracy across an extremely wide range [26,39,40,41]. In practical industrial deployments, they can easily achieve measurement ranges of tens of meters. For example, Renishaw’s XL-80 series provides a stable measurement range of up to 80 m with an accuracy of ±0.5 ppm [42], and the interferometer arms of the LIGO project, initiated by the California Institute of Technology with international collaboration, reach 4 km in length [43].

**Figure 2:**
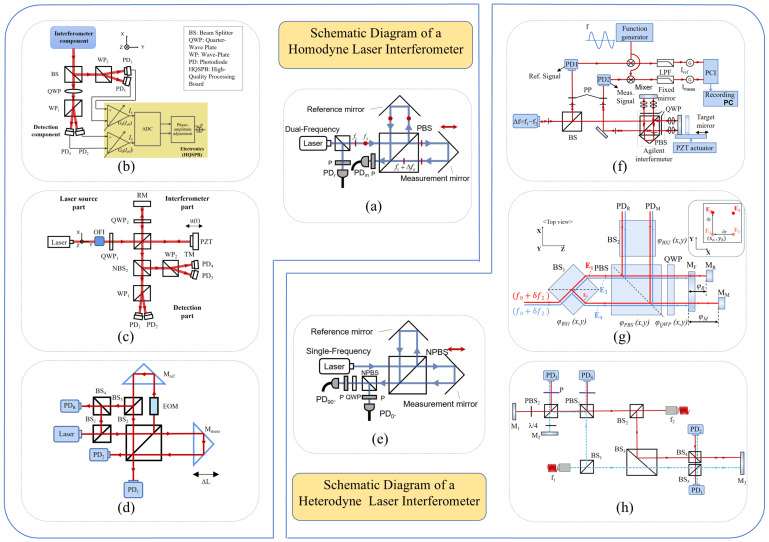
Principle diagrams of the laser interferometer. (**a**) Homodyne laser interferometer. (**b**) Nonlinear error model for a four-channel detection optical configuration. (**c**) Interferometric unit structure. (**d**) Phase-modulated dual-coaxial laser interferometer. (**e**) Heterodyne laser interferometer. (**f**) Dual-heterodyne laser interferometer. (**g**) Compact heterodyne interferometer. (**h**) Dual-path heterodyne interferometer integrating a single phase-locked loop.

The principle of a typical Michelson laser interferometer is illustrated in Figure 2a. Displacement information is obtained by analysing the phase difference between the single-frequency laser beams reflected from the reference mirror and the measurement mirror. This approach is referred to as homodyne interferometry [44,45]. Since the measurement signal contains a Direct Current (DC) component, external interference in real-world scenarios can easily cause DC drift, affecting phase demodulation. In addition, due to the non-ideal characteristics of optical components, polarization crosstalk, optical path deviations, and similar factors, the homodyne interferometry method is susceptible to periodic errors and non-orthogonal errors. The elimination and compensation of these errors are essential for improving the accuracy of homodyne interferometric measurements. Hu et al. developed a nonlinear error model for a four-channel detection optical configuration to address variations in DC bias arising from changes in the measurement beam intensity and inconsistencies in detector response [46]. Cui et al. redesigned the interferometric unit structure to eliminate non-orthogonal errors by avoiding polarization crosstalk, thereby effectively suppressing such errors [47]. Yan et al. proposed a phase-modulated dual-coaxial laser interferometer aimed at minimizing polarization-induced nonlinear and non-orthogonal errors by avoiding the use of polarization-sensitive optical elements such as the Polarizing Beam Splitter (PBS) and wave plates, thereby achieving higher precision in laser interferometry [48] (as illustrated in Figure 2b–d). Beyond the structural optimization of the optical path, several error-compensation algorithms based on parameter estimation and related approaches have also been proposed [16,49,50].

In contrast, the heterodyne laser interferometer shown in Figure 2e employs two closely spaced optical frequencies generated using dual-frequency lasers [51,52]. Unlike homodyne interferometry, which uses an amplitude-modulated signal as the measurement signal, heterodyne interferometry utilizes a frequency-modulated signal, thereby effectively suppressing the influence of DC drift. By adopting techniques such as digital phase-locked loops or digital filtering, more precise real-time phase extraction can be achieved [53,54].

Commercial interferometers using a helium–neon laser as the light source provide an optical resolution of λ/2; however, with fringe interpolation techniques, the displacement measurement resolution can be further improved to λ/2048 (approximately 0.31 nm) [55]. Nevertheless, periodic nonlinear errors persist, arising from factors such as mixed-frequency source effects and leakage in polarization components, which degrade the coherence of the interference signal and introduce additional phase variations. Most commercial laser interferometers typically exhibit periodic nonlinear errors ranging from a few nanometres to several tens of nanometres. To address this issue, some researchers have employed spatially separated polarization beams to suppress such errors [56]. For example, Joo et al. utilized two spatially separated beams with frequency differences to prevent polarization mixing, and generated the beams at distinct frequencies from a stable single-frequency light source using an acousto-optic frequency shifter [57].

In 2015, Yan et al. proposed a dual-heterodyne laser interferometer capable of simultaneously achieving high-precision measurements of both linear and angular displacements [58], as shown in Figure 2f. The system adopted a phase demodulation method based on cross-correlation analysis and implemented high-speed signal processing via a PXI-bus-based data acquisition system. Its optical configuration was designed with a high degree of symmetry, enabling the effective utilization of common-mode noise suppression, which significantly reduced low-frequency noise induced by environmental disturbances such as temperature fluctuations and vibrations. Experimental results demonstrated that at 1 Hz, the system achieved noise levels of approximately 1 pm/Hz^1^/^2^ for linear displacement and 0.5 nrad/Hz^1^/^2^ for angular displacement, showcasing exceptional resolution in the picometre–nanoradian range.

In 2020, Joo et al. developed a compact heterodyne interferometer that achieved picometre-level displacement sensitivity over a frequency range exceeding 100 MHz in air [59], as shown in Figure 2g. By adopting an optical design with spatially separated beams to maximize the common-mode optical path, the system effectively prevented frequency and polarization mixing, thereby eliminating periodic errors. Compared to commercial interferometers, the prototype exhibited a higher sensitivity of 3 pm, twice the thermal stability, and no detectable periodic errors.

In 2022, Dong et al. proposed a dual-path heterodyne interferometer that integrated a custom-built single phase-locked-loop phase meter with down-conversion techniques, thereby reducing dependence on high-sampling-rate Analogue-to-Digital Converters (ADCs) and enabling low-cost sub-nanometre displacement measurements [60], as shown in Figure 2h. Experimental results showed that the system could accurately measure displacements of approximately 2.6 nm and that the self-developed phase meter achieved superior noise suppression compared to commercial devices at sub-nanometre noise levels, demonstrating the potential of this approach for high-precision, low-cost displacement sensing.

In optical interferometry, the distance to be measured *d* is expressed as $d=\frac{\lambda }{2}\left(m+\frac{\phi }{2\pi }\right)$, where λ is the equivalent wavelength of the light source, ϕ is the interference phase wrapped between θ and 2π, and *m* is a positive integer. Due to its inherently periodic nature, the measurement exhibits a repetitive cycle, thereby only enabling incremental displacement detection and failing to meet the requirements for absolute positioning. In an incremental interferometer, the displacement increment is obtained by accumulating the phase angle ϕ as the target moves. To measure absolute distance, multiple-wavelength light sources can be used independently for measurement. Measurement light with a wavelength $\lambda_{i}$ has a phase increment $\Delta \phi_{i}$i, yielding multiple constraint equations. Combined with the condition that *m* is a positive integer, the absolute distance can be derived [61,62,63]. This method relies on precise and stable light-source wavelengths; solutions using frequency combs from femtosecond lasers have improved measurement performance [64]. Another approach uses a tunable external cavity laser diode (ECLD) to generate a continuously wavelength-scanned source, but measurement speed is limited by wavelength scanning time, making it unsuitable for dynamic measurements [65,66].

However, laser interferometry relies on wavelength, which is sensitive to environmental temperature, humidity, and pressure in non-vacuum environments; thus, laser interferometers require relatively stable operating conditions [67,68,69].

#### 2.1.2. Optical Linear Encoder

Optical linear encoders utilize scale graduations on optical devices as the reference benchmark for displacement measurement and are categorized into incremental and absolute types [70,71,72]. Compared to laser interferometers, they are easier to integrate into compact mechanisms. In particular, absolute optical encoders based on optical scales offer small size, low cost, and high robustness, making them suitable for rapid deployment in complex production environments. For example, the RSF Elektronik MS 45 optical linear encoder adopts a singlefield scanning method, with the readhead integrating subdividing electronics to achieve a resolution of 0.5 μm and a maximum measuring length of 30 m [73]. The HEIDENHAIN LC 200 series of absolute sealed linear encoders use absolute encoding combined with incremental tracks, along with an ASIC on-chip decoder, to achieve a resolution of 0.01 μm [74]. With advancements in grating interferometer research, incremental optical encoders have rapidly caught up with, and have even surpassed, laser interferometers in terms of resolution. For instance, the Renishaw TONiC series of optical incremental encoders, which employ a transmissive grating structure, offer nano-scale accuracy and a measurement speed of 10 m/s [75]; the Magnescale BL50H series of grating interferometric encoders, utilizing high-frequency heterodyne demodulation technology, achieve a maximum resolution of 6.1 pm and a response speed of up to 5 m/s [76]. For the increasingly miniaturized semiconductor industry—especially for applications such as lithography machine workpiece stage positioning—grating interferometers have become a highly popular solution. For example, Zygo’s lithography-specific position sensors are widely used for precise position and tilt measurements in lithography machines [77]. Due to these advantages, the market share of optical encoders in North America and Japan has grown year by year since 1994 [3,78], demonstrating significant value potential.

**Figure 3:**
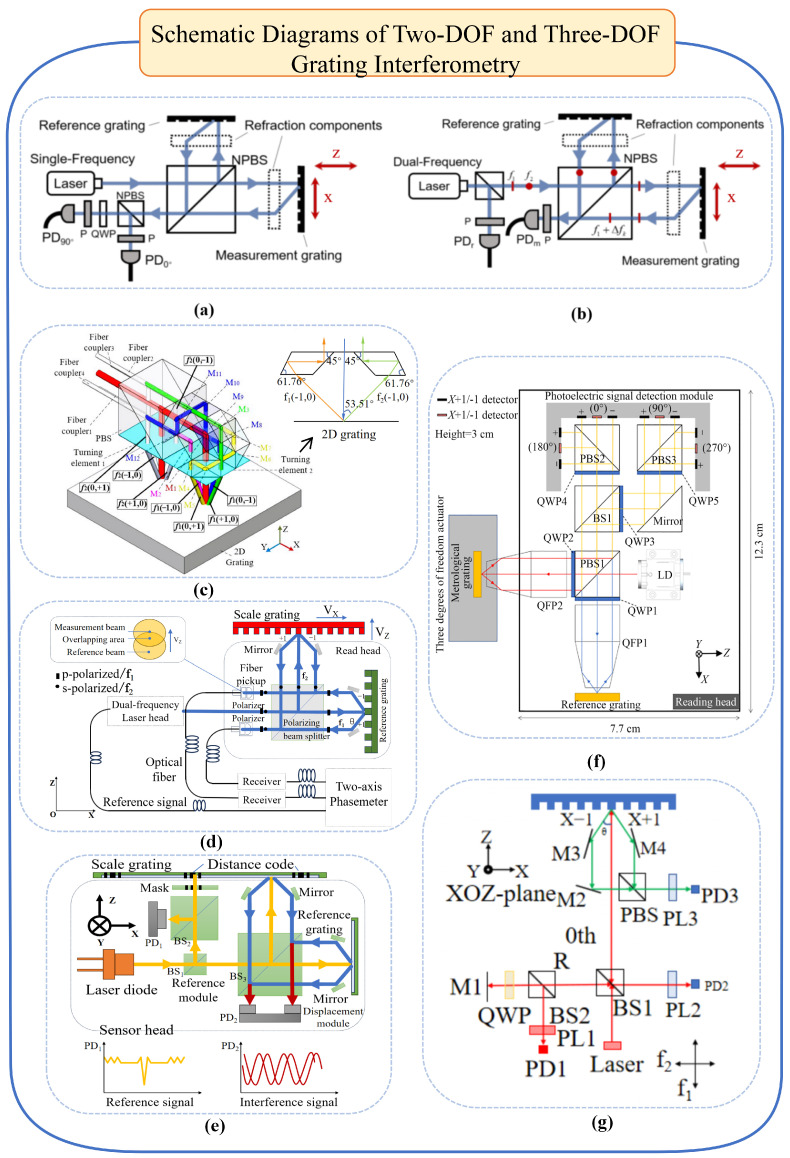
Schematic diagrams of two-DOF and three-DOF grating interferometry. (**a**) Homedyne grating interferometer. (**b**) Heterodyne grating interferometer. (**c**) Schematic diagram of high-precision, long-stroke, two-dimensional grating displacement measurement system. (**d**) Heterodyne two-degree-of-freedom system. (**e**) Absolute two-degree-of-freedom grating interferometer. (**f**) Three-DOF grating encoder based on quadrangular pyramid prismr [84]. (**g**) External heterodyne three-DOF grating interferometer.

A representative example of an incremental optical linear encoder is the grating interferometer, which employs the grating constant g as the measurement reference. Similar to laser interferometers, grating interferometers can be classified into homodyne and heterodyne types, with the measurement principle shown in Figure 3a,b. The grating interferometer is capable of measuring multiple degrees of freedom of linear displacement within a single-axis structural configuration. In contrast, owing to the limitations imposed by the laser wavelength and the requirement for beam parallelism, a laser interferometer must integrate multiple subsystems positioned around the measurement target in order to achieve multi-axis measurements [79,80,81].

Although the measurement range of grating interferometry is limited by the technical challenges of manufacturing large-sized gratings (typically at the millimetre or metre scale) and laser interferometers thus offer a broader range, grating interferometers can achieve accuracies down to tens of picometres [79,82,83], matching the performance of laser interferometers. More importantly, the measurement reference of grating interferometers is the grating constant, which is unaffected by the air refractive index. Additionally, gratings made from zero-thermal-expansion materials can resist temperature-induced effects, eliminating the need for additional calibration and enabling adaptation to more complex working environments. As a result, the market share of optical linear encoders—represented by grating interferometers—has continued to grow over the past few decades.

With the advancement of industrial automation and intelligent manufacturing, the prevalence and demand for multi-axis Computer Numerical Control equipment in industrial production continue to grow. Grating encoders are trending toward multi-axis measurement capabilities, enabling simultaneous precision positioning and high-order error motion measurement. They play a crucial role in displacement measurement, digital holography, wavefront aberration detection, phase-contrast imaging, absolute planar positioning, and optical filtering [85,86,87,88,89,90]. Classification based on the number of measurement degrees of freedom includes single-degree-of-freedom, two-degree-of-freedom, three-degree-of-freedom, and multi-degree-of-freedom systems. This section summarises the representative research on two- and three-degree-of-freedom systems. Two-degree-of-freedom measurement refers to techniques that simultaneously measure an object’s motion or positional changes along two mutually orthogonal directions within a specific plane or space. High-precision and highly stable two-dimensional displacement measurement is widely required in modern industries such as semiconductor manufacturing, micro–nanofabrication, and displacement measurement of lithography system wafer stages [91,92,93,94]. Depending on the measurement dimensions and application scenarios, two-degree-of-freedom measurement can be categorised into XY two-dimensional measurement and XZ two-dimensional measurement. XY two-dimensional measurement primarily focuses on displacement variations of objects within a two-dimensional plane. Early XY two-dimensional measurement relied on optical interferometry techniques such as the Michelson interferometer [95], which offered high precision, but was bulky. In 2008, Cheng-Chih Hsu proposed one-dimensional and two-dimensional in-plane displacement measurement based on heterodyne grating interferometry, achieving sub-pm sensitivity and enabling two-dimensional in-plane measurement using a single interferometer [87,92]. In 2022, Li, from the Changchun Institute of Optics, Mechanics, and Physics, Chinese Academy of Sciences, proposed a two-dimensional grating displacement measurement system employing dual-space heterodyne optical path interleaving. This achieved real-time acquisition of high-precision, long-stroke two-dimensional displacement information, as illustrated in Figure 3c.

XZ two-dimensional measurement focuses on object variations in both the horizontal and vertical dimensions, typically involving height and depth measurements. Consequently, non-contact measurement methods such as laser ranging or structured light are required. Laser ranging obtains distance information by measuring the flight time or phase shift of the laser beam [96], while structured light projection projects a specific pattern onto the object’s surface. A camera then captures the image, and image processing algorithms calculate the object’s height or depth information [97]. Recent developments in XZ two-dimensional measurement include the following: Gao et al. proposed a two-degree-of-freedom zero-difference grating interferometer readhead. By utilising positive and negative first-order diffraction beams from reference and scale gratings to resolve displacement signals, sub-nanometre resolution was achieved for both X and Z axes [98,99,100]. In 2014, Zhu et al. proposed a heterodyne grating interferometer system capable of simultaneously measuring in-plane displacements over hundreds of millimetres and out-of-plane displacements over hundreds of micrometres. The structure is illustrated in Figure 3d [94]. In 2017, Zhu et al. developed a novel non-contact single-point two-dimensional measurement system capable of simultaneously and independently measuring in-plane and out-of-plane displacements. This system achieved a two-dimensional displacement measurement range of 500 μm with sub-micron precision [101]. In 2019, Li et al. developed an absolute optical encoder with nanometre-level repeatability, as depicted in Figure 3e. This encoder incorporates an enhanced scale grating and a dual-probe compact readhead that are utilised, respectively, for approximate position marking and nanometre-resolution displacement measurement via grating interferometry. Experimental results indicate a repeatability of 10 nm for this encoder [102].

Three-degree-of-freedom measurement primarily extends the XYZ plane to enable multi-axis displacement measurement. Replacing the traditional Michelson interferometer plane mirror with a three-axis displacement sensor achieves nanometre-level resolution [31]. Using gratings and laser autocollimators, three-dimensional angles can also be measured simultaneously, enabling three-degree-of-freedom measurements [103]. Building upon this work, Kimura et al. proposed a three-axis optical readhead with sub-nanometre resolution for platform motion measurement. Testing demonstrated sub-millimetre resolution across all three axes [104,105]. As semiconductor wafer diameters increase, gratings exceeding 500 mm × 500 mm scales become necessary. Manufacturing large-scale gratings has proven to be prohibitively costly, and gravitational deformation compromises measurement accuracy [33]. Consequently, in 2014, Shimizu et al. enhanced the three-axis readhead by integrating a mosaic grating. Experimental validation confirmed the feasibility of the designed four-readhead optical sensor structure and mosaic grating concept, while extending the measurement range of the XY axes [84]. However, Z-axis displacement measurement in this system remains somewhat constrained, as substantial Z-displacement causes the light spot on the detector to shift, thereby weakening the measurement signal. In 2017, Lin Jie et al. proposed a three-axis grating encoder featuring nanometre resolution and a significantly expanded Z-axis measurement range, and achieved a 4 nm Z-axis displacement resolution [106].

In 2022, Li Xinghui proposed a compact, high-precision three-degree-of-freedom grating encoder based on a quadrangular prism (as shown in Figure 3f). The measurement platform leverages the self-collimation capability of miniaturised quadriconical prisms. Laser light undergoes processing through the measuring grating and optical path subdivision modules (including QWP, PBS, etc.), generating specific phase-modulated photoelectric signals to compute displacement in the X, Y, and Z directions [84]; this encoder achieves average displacement accuracy below 500 nm with a minimum error of 0.0708 percent, demonstrating miniaturisation potential. Future improvements in grating fabrication may enable millimetre-range measurement with 1‱ precision [107,108,109].

Two technical approaches exist for heterodyne three-degree-of-freedom interferometry: the first involves adding an external measurement axis to a heterodyne interferometer with one degree of freedom, such as the heterodyne grating-based interferometer proposed by Hsieh et al., which combines the advantages of heterodyne and grating interferometry to achieve nanometre resolution and millimetre displacement measurement range on a three-degree-of-freedom platform [110]; the second approach employs dual-frequency laser measurement systems. In 2021, Zhou Siyu’s team proposed a dynamic three-degree-of-freedom measurement technique based on dual-comb interferometry and a self-designed grating-based angular cube (GCC) composite sensor, capable of precisely and rapidly measuring long-range distances and two-dimensional angles [111]. In 2022, Zhu employed a reflective optical structure to achieve sub-nanometre precision in three-degree-of-freedom measurements, as illustrated in Figure 3g [110]; Li Xinghui designed a heterodyne three-degree-of-freedom grating interferometer utilising a reflective plane grating for the readhead. Leveraging a custom-built dual-frequency laser source, all three axes achieved resolutions better than 0.6 nm, with 20 s stability exceeding 5 nm. The reflective plane grating readhead design effectively mitigated Abbe error and environmental interference. This apparatus demonstrated robust testing capabilities for ultra-precision motion systems [112]. These advancements hold promise for application in advanced manufacturing and lithography fields.

**Figure 4:**
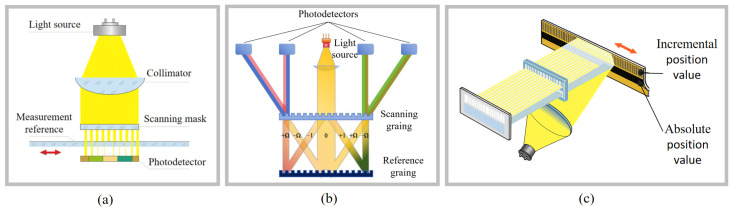
Principle diagrams of the one-dimensional optical linear encoder: (**a**) Incremental mask-type optical grating encoder. (**b**) Incremental interference-type optical grating encoder. (**c**) Absolute optical grating encoder.

Optical encoders based on grating diffraction and doppler effects exhibit excellent performance in integrated multi-degree-of-freedom measurements; however, in one-dimensional linear displacement applications, they are equally capable of achieving integrated high-precision designs. In the configuration illustrated in Figure 4a, light passes through a mask grating and a reference grating, and the periodic variation in signal intensity generated is measured using a photodetector. The relative displacement between the mask grating and the measurement reference can then be calculated from this periodic signal pattern. A further improvement, as shown in Figure 4b, utilizes symmetric differential signals produced by grating diffraction to enhance the SNR, thereby enabling higher measurement accuracy.

The aforementioned two optical encoders are both used for incremental displacement measurement, while the absolute optical linear encoders use scales with multi-track parallel codes (e.g., binary Gray codes) or serial codes (e.g., M codes) to establish a one-to-one correspondence between target displacement and unique digital codes, thereby obtaining absolute positions within the measurement range, as shown in Figure 4c. This avoids the inherent issues of incremental encoders, such as reset delays, redundant errors, and position loss upon power failure [113,114,115,116,117].

These methods have been widely applied in commercial one-dimensional grating scales. For example, the HEIDENHAIN LC 115 series employs a single-track M-code scheme for absolute position encoding, supplemented by an additional incremental track for subdivision interpolation. Based on the imaging scanning principle, the graduation pattern (with a pitch of either 20 µm or 40 µm) is optically projected onto a photosensitive sensor, where the periodic modulation of light intensity produces stable position signals, enabling absolute positioning with a resolution of up to 1 nm over a measuring length of 4240 mm [118]. In addition to the imaging scanning approach, some absolute optical encoder products from HEIDENHAIN adopt the interferential scanning principle, utilising gratings with finer pitches (e.g., 8 µm). In this case, diffracted beams originating from the fine graduation structures interfere with each other, and the resulting phase variations are converted into high-resolution sinusoidal signals [119].

Similarly, the Renishaw RESOLUTE™ series employs a single-track absolute optical scale with a non-repeating code pattern, read by a high-speed detector to deliver resolutions down to 1 nm at speeds up to 100 m/s, while maintaining high immunity to dirt and signal contamination. It supports multiple serial communication protocols (e.g., BiSS-C, FANUC, Siemens DRIVE-CLiQ), enabling direct integration into advanced motion control systems [120].

With the development of ultra-high-speed image sensors, absolute optical encoders can capture large amounts of high-resolution image data during high-speed motion. By employing high-performance image acquisition and processing devices (e.g., DSPs) and algorithms, interference from factors such as vibration, lighting changes, and surface reflections can be reduced, enabling the low-cost implementation of high measurement accuracy during high-speed motion [71]. Furthermore, encoders based on this principle can not only be used with linear scales, but also in disk encoders, enabling rotational angle measurement in certain application scenarios [114].

#### 2.1.3. Confocal Measurement Method

**Figure 5:**
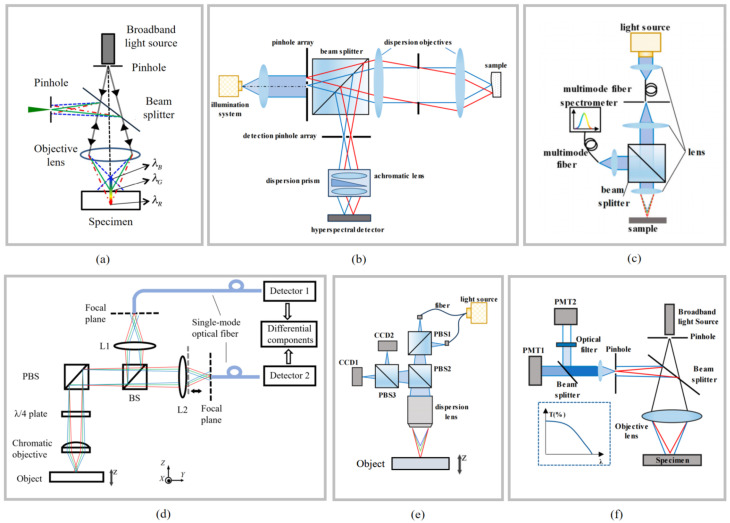
Principle diagrams of confocal displacement measurement: (**a**) Basic principle of the confocal probe. (**b**) Array-type aperture structure. (**c**) Structure using optical fibre instead of a pinhole. (**d**) Differential structure. (**e**) Spectral analysis using a CCD. (**f**) Spectral analysis based on optical transmittance.

Confocal probes are among the important measurement tools in precision metrology, widely used for precise measurement of complex three-dimensional microstructures and capable of providing high-resolution axial positioning [3,34,121,122,123]. The typical structure of a confocal probe is shown in Figure 5a. A light beam emitted by the light source is focused on the surface of the measured object through an objective lens. The reflected beam passes through a pinhole aperture at the confocal position before being detected by a detector. The distance between the objective lens and the focal plane on the surface of the measured object is derived via the wavelength–refractive index relationship.

Probes using traditional single-frequency laser sources are called laser confocal probes, which require the measurement head to move axially with high precision to focus the beam on the target surface. Their measurement performance is limited by the resolution, accuracy, and speed of the probe drive mechanism [124]. To address the limitations of monochromatic confocal probes, researchers have developed chromatic confocal probes using white light sources. Only the confocal wavelength components can pass through the pinhole aperture to be captured by the detection unit, while out-of-focus components are blocked. By analysing the spectral characteristics of the detected wavelengths, the distance between the objective lens and the surface of the measured object can be directly obtained, reducing vibration effects caused by mechanical movement and simplifying system configuration.

The size of conjugate pinholes plays a critical role in enhancing both the resolution and the signal-to-noise ratio (SNR) of confocal detectors. To enable the acquisition of multiple sampling points within a single measurement, multi-aperture configurations have been proposed by researchers. For example, Hwang et al. [125] employed a rotating disk aperture to modulate the optical frequency, thereby achieving high-contrast three-dimensional imaging. In addition, array-type aperture structures have been developed for simultaneous multi-point measurements, as shown in Figure 5b, effectively improving detection efficiency [126,127]. Beyond traditional pinhole designs, optical fibres have emerged as an ideal alternative due to their small diameter, low signal loss, and stable beam propagation characteristics [128,129]. As illustrated in Figure 5c, a single multimode optical fibre can function as both a light-transmission medium and a pinhole, thereby simplifying the optical path and facilitating modular system design.

The axial measurement range of a chromatic confocal probe is determined by the spectral range of the light source employed. To extend the chromatic dispersion range, a multi-lens assembly configuration has been proposed, achieving a maximum dispersion range of up to 30 mm [130]. However, increasing the structural complexity of the chromatic probe is unfavourable for system integration, and the use of curved lenses can degrade the SNR. Diffractive optical elements (DOEs) have therefore been introduced for generating dispersion. Light of different wavelengths passes through a DOE at varying angles, producing dispersion based on diffraction principles. Compared to refractive lenses, the focal points of longer wavelengths are closer to the focal position generated by the DOE.

On the other hand, given that an extended measurement range requires white light sources with a broader spectral bandwidth, the inherently low spatial coherence and instability of such sources impose limitations on measurement accuracy. To address this issue, an alternative approach employing a mode-locked femtosecond laser source has been proposed, which consists of a series of optical frequencies with specific modes and uniform intervals. It features highly stable spectral characteristics and high spatial coherence, making it advantageous for dispersive confocal imaging [131,132]. Mode-locked laser sources have femtosecond-level pulse widths in the time domain and high peak pulse power, which are beneficial for spectral analysis. Based on Fourier transform properties, they inherently have a broadband spectrum in the frequency domain, enabling precise measurements over a wide range [11,133]. Nevertheless, the spectrum of mode-locked laser sources is non-smooth, with extremely narrow spectral widths corresponding to individual modes. To achieve high-precision measurements over a continuous range, a spectral confocal method using supercontinuum laser sources has been proposed, which involves coupling mode-locked laser sources into nonlinear photonic crystal fibres to broaden the spectral range [134,135,136].

In addition, researchers have developed a differential dual-detector spectral confocal configuration [137,138], as shown in Figure 5d. The reflected beam passes through two confocal probes: a measurement detector positioned at the focal point, and a reference detector at a defocused position. By performing a differential operation on the two signals, the normalised intensity ratio between the outputs of the measurement and reference detectors is obtained. This effectively eliminates the influence of non-uniform spectra from mode-locked laser sources and mitigates the effects of uneven sample reflectivity.

Chromatic confocal measurement encodes the axial position using the focal wavelength of the reflected light, with its resolution being highly dependent on spectral detection and peak extraction. In this approach, a prism or diffraction grating is employed to disperse the light, and the intensity at different wavelengths is subsequently detected using photodetectors—a method that has been widely adopted in commercial spectrometers. In addition, several studies have explored alternative techniques, such as utilizing CCD sensors or measuring optical transmittance to obtain the wavelength distribution [139,140], as illustrated in Figure 5e,f. However, the resolution of these detection methods remains relatively low, and they are limited to identifying only specific spectral bands.

Confocal measurement technology is widely applied in precision manufacturing for continuous displacement and thickness measurements, and it is suitable for a variety of complex measurement scenarios, including thickness gauging of transparent multilayer structures, internal surface contour measurement in confined spaces, dynamic in-process inspection on coordinate measuring machines, and high-precision gap positioning between glass masks and substrates. Mature commercial products offer nanometre-level resolution, with probe head volumes reduced to the millimetre scale. For example, the Micro-Epsilon confocalDT IFD2410/2415 series achieves a measurement resolution of 12 nm and a measurement speed of 25 kHz, featuring compact inline controllers and the capability to measure highly inclined and transparent surfaces with thickness gauging for multi-layer substrates. The Keyence CL-3000 series adopts a white-light multi-point confocal scanning method, enabling non-contact 3D profile acquisition with up to 0.1 µm resolution and high-speed sampling for dynamic in-process inspection; its optical unit is separated from the controller, reducing installation constraints in production lines. The Precitec CHRocodile CLS line-confocal sensors integrate spectral confocal technology with a high dynamic range detection module, permitting simultaneous measurement of both high-gloss and low-reflectivity surfaces, and supporting precise thickness measurements of transparent layers in glass, polymer films, and semiconductor wafers. However, confocal measurement technology is only suitable for axial measurements: excessive surface angles or weak reflections can cause signal loss, and the measurement range is typically no more than several tens of millimetres [141,142,143,144].

#### 2.1.4. Laser Triangulation

**Figure 6:**
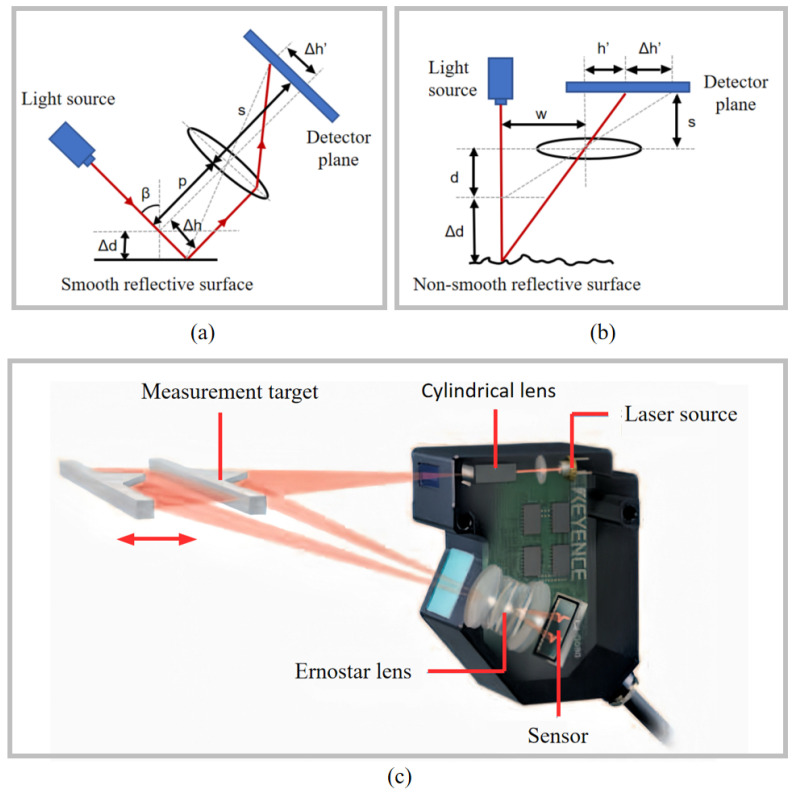
Schematic illustration of the laser triangulation principle. (**a**) Measurement setup for smooth surfaces. (**b**) Measurement setup for rough surfaces. (**c**) Laser-triangulation distance-measuring probe.

Laser triangulation sensors are optical sensors that measure distance based on trigonometric geometry principles. They are widely used in industrial measurement, contour scanning, surface inspection, and other fields—typically in industrial applications requiring fast, non-contact measurement with sub-micron resolution. Their advantage of a long working distance further extends their use in numerous industrial scenarios [145,146,147,148,149]. Figure 6 shows two configurations of laser triangulation sensors, selected based on the different reflectivity of the measured object’s surface. In principle, the laser beam is captured by the detector after undergoing specular or diffuse reflection on the object’s surface. By analysing the displacement of the reflected light spot on the detector using similar triangle relationships, the distance between the measured target and the sensor can be calculated. Figure 6a illustrates the inclined incidence scheme, chosen for measuring smooth reflective surfaces. The geometric relationship is expressed as

(1) $$\Delta d=\frac{p}{s}\Delta h^{\prime }\cos\beta$$

where β is the incident angle of the measurement laser beam on the target surface, *p* is the distance between the target surface and the lens, *s* is the distance between the lens and the detector plane, and $\Delta h^{\prime }$ is the displacement of the spot on the position-sensitive detector. Figure 6b shows the vertical incidence scheme selected for measuring non-smooth reflective surfaces. Its geometric relationship is expressed as

(2) $$\Delta d=w\cdot s\left(\frac{1}{h^{\prime }}-\frac{1}{h^{\prime }+\Delta h^{\prime }}\right)$$

where *w* denotes the distance between the laser beam and the lens axis and *s* denotes the distance between the lens and the detector plane. $h^{\prime }$ is determined by *w*, the initial position *d* of the target, and the focal length of the lens.

Commercial laser triangulation probes have achieved high speed, high integration, and typical accuracies ranging from the micrometer level to sub-micrometer precision. They are well-suited for direct and rapid deployment in complex production environments, fulfilling the requirements of fast dynamic measurement scenarios. For instance, the LJ-G Series from KEYENCE, as illustrated in Figure 6c, is capable of two-dimensional distance measurement with a linearity of 0.1% of full scale. It supports the simultaneous measurement and evaluation of up to eight features, significantly enhancing inspection efficiency, and provides stable measurement performance across a wide range of target types, without being notably affected by material properties or surface conditions [150].

The detector of a laser triangulation system typically uses image sensors such as CCD or CMOS, which locate the position of the reflected light spot via image processing algorithms. However, factors such as the local slope of the target surface, surface roughness, texture variations, and ambient light changes can introduce speckle noise, affecting measurement accuracy [151,152,153,154].

#### 2.1.5. Time-of-Flight Measurement Method

**Figure 7:**
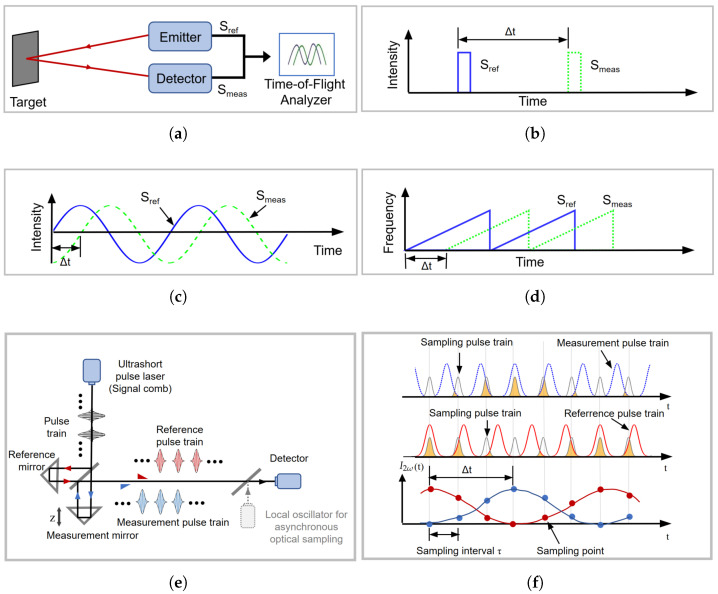
Schematic diagram of time-of-flight measurement. (**a**) Measurement structure. (**b**) Pulsed signal. (**c**) Sinusoidal intensity modulation. (**d**) Continuous frequency modulation. (**e**) Time-of-flight measurement system based on a Michelson interferometer configuration. (**f**) Sampled correlation intensity of dual-pulse train asynchronous optical sampling method.

The base unit of distance (meter, m) is defined by the speed of light in a vacuum and the base unit of time (second, s) [155]. Measuring distance by detecting the flight time of light pulses is a direct and effective approach. Traditional time-of-flight (TOF) methods use continuous-wave laser sources, modulated into periodic pulse or sinusoidal functions (Figure 7b,c), with the modulation period as the measurement reference. The time difference between the reference and measurement beams is detected to determine the flight time [156,157]. Additionally, another method adjusts the bias current injected into a diode laser to control its instantaneous optical frequency, achieving optical frequency modulation (Figure 7d). Flight time is measured by detecting the beat frequency between the reference and measurement beams, a technique referred to as frequency-modulated continuous-wave (FMCW) [158,159]. However, these modulation methods are accompanied by cyclic errors, limiting TOF measurement accuracy. Their time measurement resolution is typically at the picosecond level, corresponding to a distance resolution of up to several hundred micrometers [160]. Thus, traditional TOF methods have primarily been applied to large-scale distance measurement scenarios and are unsuitable for high-precision tasks.

As a self-modulated light source, mode-locked femtosecond pulse lasers feature extremely short and highly stable pulse repetition intervals, with pulse durations ranging from hundreds to a few femtoseconds (1 fs corresponds to a light propagation distance of 0.3 mm). In the time-of-flight measurement system with a Michelson interferometer layout (Figure 7e), the relationship between the time delay of the measured pulse sequence relative to the reference pulse sequence and the measured displacement *d* is expressed as

(3) $$d=\frac{v_{g}}{2}\left(m\cdot \tau_{r}+t_{d}\right)$$

where $v_{g}$ is the group velocity of the pulse sequence in air, $\tau_{r}$ is the pulse repetition interval, *m* is the integer number of measurement pulse sequences delayed by the integer number of repetition intervals relative to the reference pulse sequence, and $t_{d}$ is the fractional time offset.

There are three main methods for measuring the time difference between the measured pulse sequence and the reference pulse sequence. The first method utilises the optical coherence between the two pulse sequences to generate interference fringes and resolve the correlated signal $I(t_{d})$, thereby obtaining the time offset between the pulses in the time domain [161]. The relationship is expressed as

(4) $$I\left(t_{d}\right)=I_{mf}\left(0\right)+I_{mf}\left(t_{d}\right)\cos2\pi v_{c}t_{d}$$

where $I_{mf}\left(t_{d}\right)$ corresponds to the correlation intensity between the measurement signal and the reference pulse signal envelope, and $v_{c}$ is the carrier frequency of the pulse.

Due to the highly stable carrier frequency and phase of mode-locked lasers, the interference fringes have a long coherence length, enabling high-contrast interference signals, even when the two pulses are not perfectly aligned. This method achieves an accuracy better than half an optical wavelength (<λ/2); with highly stable lasers, the ranging range can extend to hundreds or even thousands of kilometres (1500 km) [162,163,164].

The second method is second harmonic (SH) correlation measurement, a time-of-flight ranging technique that generates non-fringe-resolved autocorrelation signals [165,166]. When the measurement and reference pulse trains are incident on a nonlinear crystal simultaneously, an SH pulse is generated at their overlap region [167]. The correlation intensity signal of the SH sub-pulse, $I_{2\omega }\left(t_{d}\right)$ is expressed as

(5) $$I_{2\omega }\left(t_{d}\right)=\int I_{\omega ,M}\left(t\right)I_{\omega ,R}\left(t+t_{d}\right)\;dt$$

where $I_{\omega ,M}\left(t\right)$ and $I_{\omega ,R}\left(t\right)$ are the instantaneous intensities of the measurement pulse sequence and reference pulse sequence, respectively.

To enhance sensitivity and resolution, a balanced optical cross-correlator (BOC) is employed [168]. In the BOC, the two pulses are polarised orthogonally and incident onto a type-II phase-matched nonlinear crystal, such as a periodically poled KTiOPO4 (PPKTP) crystal [169]. Within the crystal, the orthogonally polarised pulses have different group velocities, leading to the generation of two SH sub-pulses in the forward and backward paths, respectively. The time delay between these two sub-pulses is determined by the difference in group velocities of the two pulse sequences. By inputting them into a balanced photodetector for differential operation, a differential signal is obtained that exhibits linear characteristics within a certain range, enabling highly sensitive measurements of time delay while effectively suppressing common-mode noise. Experiments have shown that BOC can achieve attosecond-level resolution, with ranging accuracy of 1.1 nm (1-s average), 2.7 nm (100 ms), and 8.7 nm (10 ms), independent of the measured distance [168,170,171].

The third method, dual-frequency comb ranging interferometry, is an asynchronous optical sampling (ASOS) approach [172,173,174] (Figure 7f). The repetition frequency fl of the detection pulse sequence generated by the local asynchronous mode-locked laser source has a slight deviation from the repetition frequency fr of the reference pulse sequence. This detection pulse samples the measurement and reference signals, yielding periodic correlation signals (as shown in the figure). This amplifies the fractional time offset between the measurement and reference pulses into the time offset between their respective correlation signals with the local detection signal. Alternatively, directly interfering the detection and measurement signals, followed by Fourier and Hilbert transforms, can extract the carrier frequency and phase to calculate distance [175,176,177,178]. Despite its significant advantages in accuracy and speed, dual-comb ranging is limited by system cost, primarily due to the requirement for two highly stable mode-locked lasers [177].

### 2.2. Angular Displacement Measurement

In high-precision mechanical motion systems, a key factor affecting the accuracy of linear displacement measurement is the Abbe error—a phenomenon in which any small angular error is magnified into an additional linear displacement error when there is an offset between the measurement reference line and the actual axis of motion, with the magnitude of the error proportional to the offset distance. For large-scale equipment such as multi-degree-of-freedom platforms, precision machine tool worktables, coordinate measuring machines, and semiconductor manufacturing systems, even microradian-level tilt or rotational deviations can translate into nanometer- or sub-micrometer-scale linear measurement errors when long Abbe arms are involved. Such displacement deviations induced by angular errors not only reduce machining accuracy, but also impair the reliability of geometric error compensation, position closed-loop control, and quality monitoring [179].

Angular displacement can be classified into two fundamental types: Rotating: Rotation about a fixed axis, as in the positioning of rotary axes in machine tools or the angular feedback of rotary tables; Tilting: The change in an object’s orientation relative to a reference plane, as in optical platform flatness adjustment or mirror alignment. For different application tasks, engineering practice requires selecting the most appropriate measurement method to ensure that angular errors are monitored and compensated in real-time, thereby eliminating or significantly reducing the influence of the Abbe error.

Table 2 provides a comparison of the various optical angular displacement measurement techniques employed in advanced manufacturing in terms of their performance and application scenarios. This section introduces optical angular displacement measurement techniques applicable to precision manufacturing from the perspective of their underlying principles, covering both mature commercial solutions and promising technologies with strong potential for future adoption.

#### 2.2.1. Rotary Encoder

**Figure 8:**
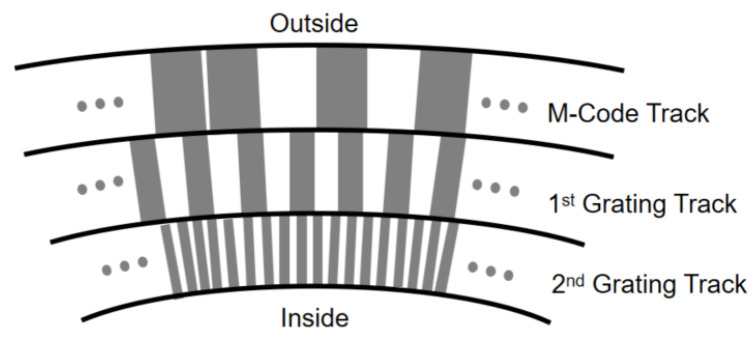
Absolute rotary encoder with incremental tracks.

The rotary encoder illustrated in Figure 8 is a widely used optical angular sensor in industrial manufacturing and represents a mature solution for measuring rotational angles. Its measurement principle is identical to that of linear encoders: both read encoded or grating patterns on the disk surface as measurement scales to achieve incremental or absolute angle measurement [180,181,182,183]. Extensive structural and algorithmic optimizations have been conducted on rotary encoders [184,185,186,187], and mature, low-cost commercial products are now available. For example, Renishaw’s RESOLUTE/RESA series of absolute angle encoders support 32-bit absolute resolution, with an angular resolution of up to 0.036 arcseconds [120]. Meanwhile, Renishaw’s REXM20/TONiC series of optical incremental encoders achieve an equivalent linear resolution of 1 nm, corresponding to approximately 0.01 arcseconds for a ring diameter of 100 mm [188]. However, rotary encoders can only be applied to fixed rotating shafts, restricting their application scenarios.

#### 2.2.2. Autocollimator

**Figure 9:**
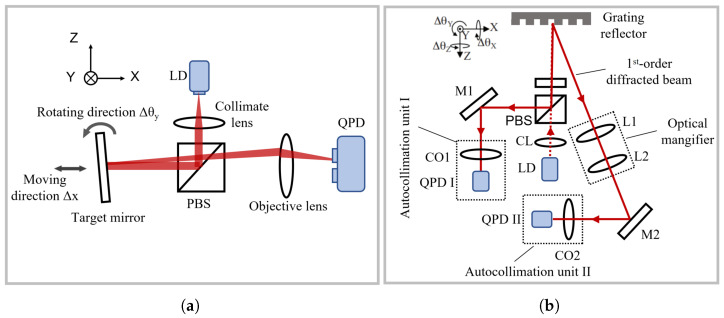
Laser autocollimator for multi-axis angle measurement. (**a**) Two-axis laser autocollimator. (**b**) Grating reflector-based three-axis laser autocollimator. (LD: Laser Diode; CL: Collimate Lens; QWP: Quarter wave plate; PBS: Polarizing beam splitter; CO: Collimator objective; M: Mirror; L: Lens).

An autocollimator measures the angular displacement of a target by detecting the deviation of a measurement beam reflected from the target surface. It is particularly well-suited for measuring minute tilt angles and is often integrated with linear displacement measurement schemes to enable multi-degree-of-freedom measurements. A typical configuration is shown in Figure 9a. Commercial autocollimators currently available can be used for linear levelling, angular deviation measurement, flatness inspection, dishiness inspection, and rotational accuracy checks, among other tasks. For instance, the ELCOMAT HR electronic autocollimator from MÖLLER-WEDEL OPTICAL GmbH achieves a resolution of up to 0.001 arcsecond with a measurement range of approximately ±150 arcseconds [189], while Nikon’s 6B-LED and 6D-LED autocollimators offer a resolution of 0.5 arcseconds and a measurement range of ±30 arcminutes [190]. This method can be applied to scenarios without a fixed rotation axis, providing a flexible approach to angular displacement measurement suitable for diverse applications. Notably, in multi-degree-of-freedom measurement tasks, autocollimation is one of the most commonly used methods for angular displacement measurement. There are typically two approaches to detect the light spot position: using an image sensor (e.g., CCD) to capture changes in the spot position [189], or using a four-quadrant photodetector (QPD) [191] to determine changes in the spot’s centre of mass by analysing energy distribution variations within the spot. Figure 9b shows the structure of a three-axis laser autocollimator [31]. By measuring diffraction at the grating surface, the zero-order diffracted light is insensitive to angular displacement of the target around the Z-axis, while the first-order diffracted light is sensitive to the three-axis angular displacement of the grating reflector. Adding two additional QPDs enables measurement of three-axis angular displacement.

#### 2.2.3. Interferometric Methods

**Figure 10:**
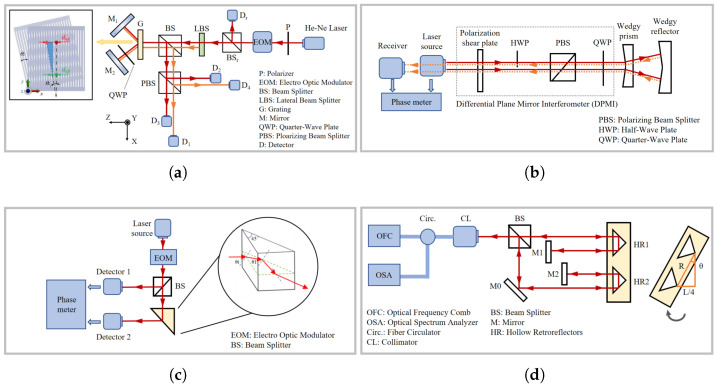
Interference-based angular displacement measurement methods. (**a**) Interferometer using double-diffraction grating for displacement and angle measurement. (**b**) Roll-angle measurement system schematic. (**c**) Angle sensor based on total internal reflection phase shift. (**d**) Optical frequency comb interferometer for angular displacement measurement.

Interferometric angular displacement measurement methods exploit the wave nature of light to extract angular displacement information by measuring the phase difference between a measurement beam and a reference beam. Since the measurement reference is the wavelength of light, these methods inherently offer exceptionally high measurement accuracy. Chen et al. proposed an interferometric measurement scheme applicable to both single-axis displacement and angular measurements [192], as shown in Figure 10a. A dual-frequency laser modulated by an electro-optic modulator (EOM) is split into two beams (L1 and L2) by a lateral beam splitter (LBS), which then pass through a transmission grating from different positions. For either beam, the ±1st-order diffracted light from the grating is reflected by the optical system and passes through the grating again, undergoing secondary diffraction. The resulting ±1st-order diffracted light is combined and detected by a pair of photodetectors (PDs). Similarly to the principle of the heterodyne interferometer for linear displacement measurement introduced in Section 3.1.2, analysing the phase of the signals detected by the PDs yields the local approximate linear displacements at two positions on the grating caused by its rotation around the Z-axis (as shown in the figure). The angular displacement of the grating around the Z-axis can then be derived from the geometric relationship. Building on this work, Hsu et al [193] proposed a coplanar dual-diffraction interferometric system for displacement and angle measurement, achieving resolutions of approximately 3 nm for displacement and 33.7 nrad for angle.

The aforementioned angular displacement interferometric systems first measure local linear displacements and then calculate the angular displacement. Shi et al. proposed a roll angle measurement system based on a differential plane mirror interferometer (DPMI) [194], as shown in Figure 10b. This system employs a heterodyne measurement principle, using wedge prisms and wedge reflectors to construct a coaxial optical system that generates diagonally symmetric dual-frequency beams. The phase information carried by the optical path difference enables roll angle measurement. Experimental results showed an average deviation of less than 5 μrad and a residual error of less than 1 μrad in nano-scale positioning systems, verifying the feasibility of this system.

Additionally, some interferometric methods are variants of non-interferometric approaches. Chiu et al. proposed a novel optical angle measurement method based on total internal reflection heterodyne interferometry (TIRHI) [195], as shown in Figure 10c. This method achieves angle detection by measuring the phase difference between s-polarised and p-polarised light during total internal reflection. Its advantages include phase difference insensitivity to light intensity, strong environmental adaptability, good anti-interference capability, and a simple, compact optical path that is easy to adjust. This method achieves high resolution ($8\times 10^{-5}$ degrees) and a wide measurement range (10 degrees), making it highly valuable for precision measurement applications.

The aforementioned interferometric methods are limited by signal periodicity, and can only be applied to incremental angular displacement measurements. In contrast, methods using optical frequency combs can utilise the broad spectral properties of frequency comb sources for dispersive interferometry, enabling high-precision absolute angular displacement measurement. In the optical frequency comb interferometric system shown in Figure 10d [196], the frequency comb pulse sequence passes through a dual-arm Michelson interferometer. To avoid dispersion-induced measurement errors, a hollow corner cube prism is used instead of a solid one as the target reflector. When the target mirror rotates, the optical path difference between the two beams passing through HR1 and HR2 changes, which is reflected in the combined interference spectrum. Analysis of this spectrum using an optical spectrum analyser (OSA) yields the displacement of HR2 at the end of the cantilever. The cantilever tilt angle θ can be expressed as

(6) $$\theta =\arcsin\left(\frac{L}{4n_{g}R}\right)$$

where *R* is the length of the sine arm, representing the fixed spacing between two resonance peaks, *L* represents the path difference between the two interference arms, and $n_{g}$ is the group refractive index of air.

#### 2.2.4. Emerging Angle Measurement Methods

Beyond the aforementioned three categories of optical angular displacement measurement techniques—rotary encoders, autocollimators, and interferometric methods—several emerging approaches have been proposed in recent years to meet the increasing demands of advanced manufacturing, particularly for scenarios requiring novel sensing principles, extended measurement ranges, or enhanced system integration. Although many of these methods are still in the early stages of research and development, they show considerable potential for future application, leveraging advances in nonlinear optics, optical frequency combs, computational imaging, and intelligent image processing. Two representative examples, namely second-harmonic generation (SHG) based methods and vision-based methods, are introduced in this subsection.

**Figure 11:**
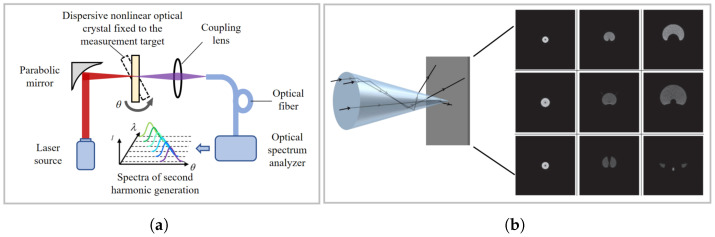
Schematic diagrams of two angle measurement methods. (**a**) An optical frequency domain angle measurement system based on second-harmonic generation. (**b**) A vision-based method and images generated after passing through an axicon at different incident conditions.

The SHG method, shown in Figure 11a, utilizes the characteristic of optical frequency combs—their spectral components have precise, definite equal frequency intervals. A beam from the optical frequency comb passes through a nonlinear material (e.g., a second harmonic crystal) fixed to the measured object, with its frequency doubled via second harmonic generation. Analysing the frequency components of the second harmonic signal using a spectrometer yields the rotational angle information of the measured object [197].

The visual method obtains angular displacement information by leveraging changes in the received image caused by angular variations in optical components [198,199,200,201]. As shown in Figure 11b, rich information carried by the shape of the light spot after refraction through a conical or converging lens is used to achieve simultaneous angle measurement [202]. Currently, the accuracy of such methods depends on image feature extraction capabilities, remaining at the sub-degree level (relatively low). However, with advancements in visual detection algorithms—especially the introduction of deep learning methods—visual methods have shown promising potential [203,204,205,206].

### 2.3. Integrated Linear and Angular Displacement Measurement

Multi-degree-of-freedom measurement extends XYZ three-axis displacement measurement by incorporating angle measurement, enabling the simultaneous characterisation of translational and rotational motions. In fields such as high-precision robotic assembly systems, six-degree-of-freedom components play a vital role. High-precision, real-time six-degree-of-freedom displacement measurement is also key to achieving precise attitude control for XY and XYZ platforms [80,207,208,209]. Future development directions for multi-degree-of-freedom grating encoders encompass the miniaturization of optical modules, the adoption of intelligent signal processing techniques, and the integration of hybrid sensing technologies [208,210,211,212]. The definition of grating interferometry measurement is more suitable for six-degree-of-freedom measurement [213]. Based on homodyne and heterodyne interferometry, multi-degree-of-freedom measurement systems typically have complex structures. They are mainly divided into two types: those that use a single optical head, and those that use multiple optical heads.

**Figure 12:**
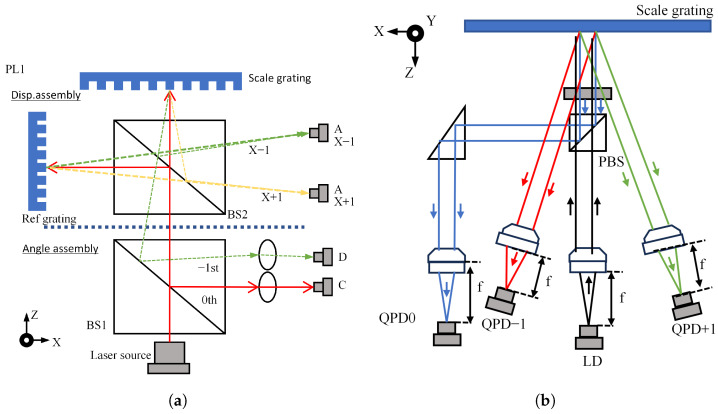
Three-DOF grating interferometry illustration. (**a**) Three-DOF grating encoder based on quadrangular pyramid prism. (**b**) External heterodyne three-DOF grating interferometer.

Single-optical-head multi-degree-of-freedom grating interferometry is based on a standard linear displacement measurement device and integrates an additional angle sensing component. By introducing an angle measurement and control module into the same optical path as the device used to measure linear motion, it is possible to simultaneously detect minute changes in displacement and angle. By extending the three-degree-of-freedom measurement method, Gao et al [31]. developed an autocollimator capable of detecting three-axis angular error motion on a precision platform, achieving a resolution of 0.01 arcseconds in angular motion across all axes. As a representative advancement in single-head grating interferometric measurement devices for multi-depth-of-field measurement, Li et al. [32] developed a multi-axis surface encoder capable of measuring six degrees of freedom of translational and angular motion, with a schematic diagram shown in Figure 12a. It uses a 405 nm blue semiconductor laser tube with a compact sensor head and planar grating to enhance motion detection. This configuration reduces Abbe error and mitigates environmental effects, achieving linearity exceeding 80 μm and stability of 5 nm within 300 s. This performance makes it an ideal choice for multi-axis ultra-precision positioning in nano-scale science and technology applications. Subsequent innovations focused on improving resolution and measurement size, such as the multi-dimensional grating interferometry system proposed by Hsieh in 2021 for evaluating grating period error [214], as shown in Figure 12b. In 2022, an absolute high-precision and multi-degree-of-freedom grating encoder [215] was developed, using a four-degree-of-freedom absolute grating encoder with three-axis attitude and Z-axis position. By fixing the read head and moving the grating reflector, sub-arcsecond and sub-micrometre accuracy was achieved.

**Figure 13:**
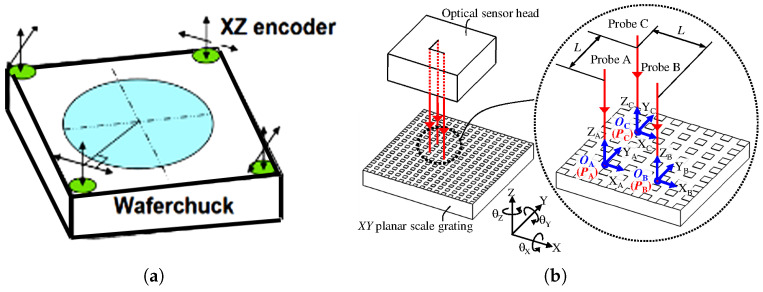
Schematic diagram of multi-optical-head multi-DOF interferometric measurement method. (**a**) ASML’s multi-optical head solution. (**b**) Comparison of optical path lengths between laser interferometry and grating interferometry.

Multi-optical-head multi-degree-of-freedom grating interferometry relies on linear displacement measurement modules placed at several different positions. By recording the relative displacement of each module, linear and angular changes can be inferred. The development path of multi-optical heads can be summarised as follows: In 2009, ASML proposed a multi-optical head scheme, establishing a multi-channel integration foundation [32], Figure 13a, for wafer stage positioning in lithography equipment. In 2010, a comparison of laser and grating interferometer optical path lengths provided a basis for optimising measurement stability in multi-optical head systems [214]. In 2014, Li Xinghui employed a multi-optical head with a right-angle arrangement [216], mounting a large XY planar grating on the workbench. The multi-probe optical sensor head was aligned with the calibrated grating to perform six-degree-of-freedom measurements of the moving platform’s planar motion. As shown in Figure 13b, this enhanced both spatial coverage and measurement accuracy.

**Figure 14:**
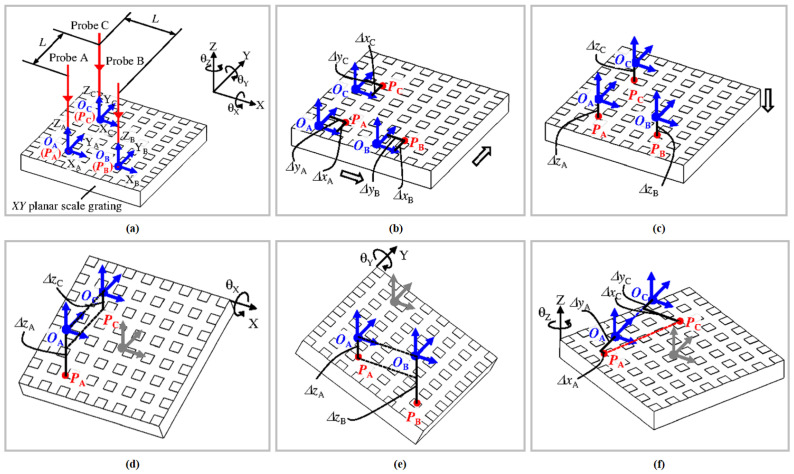
The principle of measuring six-degree-of-freedom (6-DOF) planar motions is illustrated as follows: (**a**) Initial state in the absence of any motion. (**b**) Translational motions Δx and Δy. (**c**) Translational motion Δz. (**d**) Angular error motion $\theta_{X}$. (**e**) Angular error motion $\theta_{Y}$. (**f**) Angular error motion $\theta_{Z}$ [216].

Figure 14 illustrates the working principle of a multi-probe surface encoder for measuring six-degree-of-freedom (6-DOF) planar motions. In the initial state shown in Figure 14a, the scale grating exhibits neither translational nor angular error motions. When the scale grating undergoes translation along the X-, Y-, and Z-axes, as shown in Figure 14b,c, the three-axis displacements relative to the origins $O_{A}$,$O_{B}$, and $O_{C}$ can be simultaneously obtained from the output values Δx, Δy, and Δz of the three probes (A, B, and C). Based on these measurements, the translational motions of the scale grating along the three directions (Δx, Δy, and Δz) can be derived as follows:

(7) $$\Delta x=\frac{\sum_{i=A}^{C}\Delta x_{i}}{3}$$

(8) $$\Delta y=\frac{\sum_{i=A}^{C}\Delta y_{i}}{3}$$

(9) $$\Delta z=\frac{\sum_{i=A}^{C}\Delta z_{i}}{3}$$

As shown in Figure 14d, compared to the initial state, an angular error about the X-axis leads to a differential change in the Z-direction displacement outputs of $P_{A}$ and $P_{C}$. As illustrated in Figure 14e, an angular error motion about the Y-axis similarly causes a discrepancy in the Z-direction displacement outputs between $P_{B}$ and $P_{A}$. Therefore, a small amount of secondary angular error can be expressed as

(10) $$\theta_{X}\approx \frac{\Delta z_{A}-\Delta z_{C}}{L}$$

(11) $$\theta_{Y}\approx \frac{\Delta z_{B}-\Delta z_{A}}{L}$$

On the other hand, angular error motion may also cause variations in the X- and Y-direction displacement outputs, as shown in Figure 14f. Accordingly, θ is approximately given by

(12) $$\theta_{Z}\approx \frac{\sqrt{{\left(\Delta x_{A}\right)}^{2}+{\left(\Delta y_{A}\right)}^{2}}-\sqrt{{\left(\Delta x_{C}\right)}^{2}+{\left(\Delta y_{C}\right)}^{2}}}{L}$$

In 2019, a multi-optical-head arrangement scheme for lithography wafer stage positioning was adopted, featuring multiple distributed optical heads. These optical heads are arranged in a two-dimensional grating around the wafer stage, with each optical head independently collecting local displacement information. By mathematically fusing the measurement data from all optical heads, the overall translational and rotational motion of the wafer stage is analysed. FPGA parallel processing technology is employed to achieve high-speed data fusion, meeting the dynamic response requirements of lithography equipment (response time < 1 ms) [217]. Lee et al. [109] developed a six-degree-of-freedom optical encoder system that reads grating displacement information via multiple optical heads and employs fusion algorithms to calculate six-degree-of-freedom motion errors. The optical heads are distributed across different regions of the grating, separately collecting error signals for X/Y/Z direction translations and three rotational directions. The angular error resolution is below 0.03 arcseconds, and the displacement resolution is 0.4 nm. Validation results show negligible deviation from high-precision self-collimators, making it suitable for ultra-precision machine tools, semiconductor wafer stages, and other applications. However, this design uses different optical paths for different measurement targets, leading to inconsistent environmental responses, which hinders high-precision real-time dynamic measurement. Researchers are also exploring strategies to reduce crosstalk errors, such as Matsukuma et al., who reduced crosstalk by improving and optimising the sensor head [218].

## 3. High-Precision Surface Characterization in Advanced Manufacturing

In advanced manufacturing, surface topography plays a crucial role in determining the functional performance and service life of products. Characteristics such as surface roughness, waviness, and micro- and nano-scale features not only affect assembly accuracy and tribological behaviour, but also have a significant impact on the imaging quality of optical components, the electrical properties of semiconductor devices, and the fatigue strength of aerospace parts. High-precision surface topography measurement enables engineers to monitor and optimize process parameters in real-time, detect defects at an early stage, and thus improve both manufacturing quality and consistency. Therefore, surface topography measurement is not only a key link in quality control, but also a fundamental driving force for the advancement of modern manufacturing technologies.

This section categorizes surface topography measurement into large-scale and micro-scale domains and offers a comprehensive overview of the mainstream methods in each.

### 3.1. Large-Scale Freeform Surface Metrology

#### 3.1.1. Synthetic Aperture Interferometry

**Figure 15:**
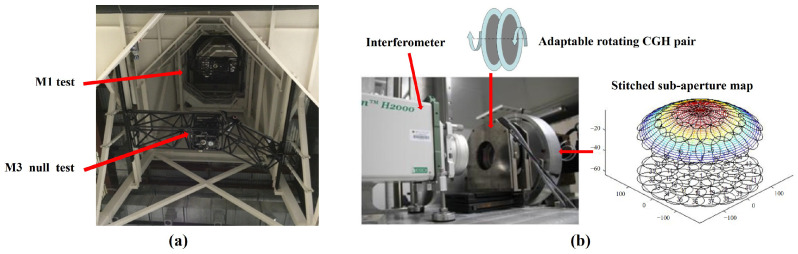
Interferometric configurations for high-precision optical surface testing. (**a**) LSST optical test setup at the University of Arizona, with M3 and M1 nulling interferometry units mounted on separate bridges. (**b**) Sub-aperture interferometry system using a multi-axis platform and rotating CGH pair for convex surface measurements, enabling full-aperture reconstruction after system calibration.

Null interferometry is a widely adopted standard method for testing precision optical components. This technique evaluates surface error by comparing the actual wavefront generated by the unit under test (UUT) with a theoretical reference wavefront. Such tests are typically implemented using computer-generated holograms (CGHs) or traditional null optics such as Offner lenses. The interferometric system is designed based on an ideal model so that only deviations from the design surface are measured, offering extremely high sensitivity and accuracy. As shown in Figure 15a, in the testing system for the Large Synoptic Survey Telescope (LSST), the 8.4 m primary mirror and tertiary mirror are measured by interferometers installed at different levels of a vertical test tower [219]. Although interferometry generally has a limited measurement range compared to non-null deflectometric methods, it provides superior resolution and stability near the nominal shape, making it ideal for final acceptance testing of optical components or systems. To address the demands of complex freeform surface testing, reconfigurable null interferometers have been proposed in recent years [220]. These systems incorporate tunable elements, such as Zernike phase plates or adjustable prisms, to flexibly adjust the wavefront and achieve local null conditions across different sub-apertures [221,222]. As shown in Figure 15b, a system combining a multi-axis platform with a rotatable CGH enables the high-precision interferometric measurement of large convex mirrors, resulting in a complete stitched surface map [223].

In the testing of complex aspheric or freeform optics, using CGHs as null optics has become a conventional approach. CGHs are fabricated with high-precision lithographic techniques derived from semiconductor manufacturing, enabling accurate wavefront control. For instance, in the final figuring phase of the Giant Magellan Telescope (GMT) primary mirror segments, a CGH-based interferometric system is used for precise error correction and verification [224]. This system combines two fold spheres with a CGH to generate the desired wavefront and achieve surface error control at the level of 20 nm RMS [225]. In addition, CGHs can be designed with integrated alignment patterns, which are co-written with the main hologram to ensure consistency and minimize additional cost [226].

Instantaneous interferometry enables the acquisition of complete interferometric data within a very short time window, making it suitable for testing environments subject to disturbances or vibration. This single-shot method records multiple phase-shifted interference patterns simultaneously within a single frame, allowing for accurate tracking of dynamic wavefront changes. For example, in the dynamic optical testing of the James Webb Space Telescope (JWST), a high-speed interferometer employing polarization-encoded phase shifting achieves imaging rates of 1 kHz at 720 × 720 pixels [227], as shown in the figure. This capability allows for the real-time measurement of structural responses under dynamic excitation, supporting the construction of system transfer function models.

Sub-aperture stitching interferometry is an advanced measurement technique well-suited for large-scale or freeform optical components. It reconstructs the full surface by acquiring and combining interferometric data from multiple local regions. Each sub-aperture is typically equipped with a dedicated CGH or null optic to create localized null conditions. This method is particularly effective for steep convex or freeform optics, such as high-speed telescope secondary mirrors, which are difficult to measure with full-aperture coverage. During measurement, either the interferometer or the test object is moved to scan across the entire surface. Sub-apertures can be designed as circular, annular, or elliptical zones depending on the geometry of the test surface [228,229]. Stitching is achieved through the overlap of adjacent regions or the use of alignment marks, while modal or zonal fitting algorithms are applied to reduce stitching errors [220,230].

#### 3.1.2. Structured Light Techniques

The earliest interference-based fringe projection system was proposed by Rowe and Welford in 1967 [231]. Since then, a variety of interferometric configurations have been developed to generate high-quality fringe patterns. Common systems include Michelson interferometers for high-frequency fringes [232,233,234], fibre interferometers for enhanced compactness [235,236,237], and Lloyd’s mirror setups [238]. By incorporating multi-wavelength sources [239,240,241], acousto-optic frequency shifting techniques [233,242], or tuning geometric parameters [238], researchers have achieved flexible multi-frequency fringe modulation. Phase-shifting can be implemented via acousto-optic deflectors (AODs) [233,243], piezoelectric transducers (PZTs) [237,241], or mechanical actuators [238].

**Figure 16:**
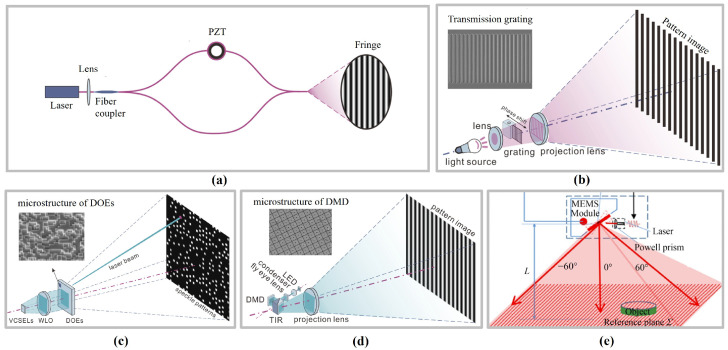
Typical structured-light system. (**a**) Schematic of interference-based fringe projection. (**b**) Schematic of physical-grating projection. (**c**) Schematic of DOEs projection. (**d**) Digital light-processing projection technique. (**e**) MEMS pattern-projection system.

Fibre interferometric systems are widely used in practical applications due to their compactness and modulation flexibility. As shown in Figure 16a, a highly coherent He-Ne laser diode is used as the source, with coupling optics and fibre couplers to split the beam into two arms of a Mach–Zehnder interferometer. A PZT-wrapped fibre enables phase modulation in one arm. The two beams recombine under far-field and near-axis conditions to form Young’s interference fringes. Phase shifting is achieved by stretching the fibre using the PZT actuator.

Advantages of interference-based fringe projection systems include continuous spatial fringe distribution, ideal sinusoidal intensity profiles, and a large depth of field. However, they also have notable limitations: relatively complex and bulky systems, higher overall cost, and sensitivity to environmental conditions such as temperature and vibration.

Unlike digital gratings, physical gratings are tangible components that are typically fabricated on glass substrates via mechanical ruling, photolithography, or embossing. Figure 16b shows the microstructure of a typical Ronchi grating. Before the digitization of structured light, physical gratings dominated fringe projection, especially throughout the 1990s. Ronchi gratings are periodic binary structures with square-wave transmittance. Although sinusoidal fringe patterns yield higher measurement accuracy when combined with phase-shifting algorithms, manufacturing sinusoidal-transmittance gratings is more challenging. Thus, binary gratings are often used, and the system’s optical response approximates a sinusoidal profile [244,245]. Additional sinusoidal shaping can be achieved via intentional defocusing [246,247,248].

In a typical 3D imaging system, the grating is illuminated by a collimated incoherent source (e.g., halogen or tungsten lamps), and the pattern is projected onto the target surface via projection optics. Incoherent sources reduce speckle effects and expand measurement range, although they decrease ambient light robustness. Phase shifting is introduced by mounting the grating on a displacement stage driven by a precision stepper motor. However, a key limitation is the fixed grating frequency, which prevents temporal phase unwrapping for improved decoding. Advantages of physical grating projection include generation of spatially continuous fringe patterns, and high-quality projections for static and high-precision tasks. Disadvantages include bulky system structure, high power consumption, relatively high cost, and lack of dynamic pattern modulation.

Diffractive Optical Elements (DOEs) are micro-structured devices based on wave optics principles that are capable of shaping laser beams via diffraction and interference to produce specific intensity patterns in the far field or focal plane. Through precise design and microfabrication, DOEs can create highly customized light field distributions and are widely used for beam shaping, splitting, and diffusion. In spatial structured-light 3D imaging, DOEs often generate pseudo-random dot patterns, commonly referred to as speckle or dot arrays, thus acting as multi-point beam generators.

With the advancement of nano fabrication technologies, 2D diffractive structures can now be mass-produced at micron or nanometre scales via lithography and etching, as illustrated in Figure 16c. This has enabled their integration in both consumer and industrial 3D imaging devices. Typically, DOEs are paired with Vertical-Cavity Surface-Emitting Lasers (VCSELs) and wafer-level optics (WLO) to form compact dot projection modules. The laser beam is first collimated and expanded by the WLO, then shaped by the DOE’s microstructures to form the desired dot pattern and project it onto the target surface.

A major advantage of DOE systems is their high optical efficiency, as most light can be directed into predefined diffraction orders. Ignoring internal losses, theoretical efficiency can exceed 90% [249,250]. Additionally, the compact “VCSEL + WLO + DOE” design makes it ideal for embedded modules in mobile devices such as smartphones and tablets. Advantages of DOE-based projection include low fabrication cost suitable for large-scale production, an ultra-compact structure ideal for embedded systems, high diffraction efficiency and energy utilization, and a large depth of field, supporting a wide imaging range. Limitations include fixed pattern design with no programmability, and sparse dot distribution and limited resolution, making them insufficient for high-precision 3D reconstruction.

Digital light modulation technologies generate and project optical patterns using programmable control of individual pixels, unlike analogue methods based on physical gratings. Representative techniques include Liquid Crystal Displays (LCD), Digital Light Processing (DLP), and Liquid Crystal on Silicon (LCoS) [251,252]. Among them, DLP is regarded as the most mature and widely used structured light modulation approach [253,254,255].

Introduced by Larry Hornbeck at Texas Instruments in 1987, DLP was commercialized in 1996 and entered the 3D imaging domain by 1998 [256,257]. Due to its compact optical structure, superior grey-scale linearity, and high image quality, DLP has gained broad acceptance in fringe projection applications.

As shown in Figure 16d, a typical DLP projection system uses an LED light source, shaped and homogenized by condenser and fly-eye lenses [258,259]. The light is then reflected by a Digital Micromirror Device (DMD), deflected by a Total Internal Reflection (TIR) prism, and projected onto the object surface. The DMD consists of an array of micro-mirrors that toggle between “on” and “off” states: mirrors in the “on” state reflect light into the projection lens to create bright pixels, while those in the “off” state divert light away, rendering dark pixels. Grey-scale modulation is achieved through high-speed switching, integrating pixel brightness over time. As a result, DLP systems are ideal for binary patterns with high frame rates, but limited speed for grey-scale projections. Advantages of DLP-based projection include pixel-level precision and high programmability, high-contrast and high-quality pattern projection, and excellent grey-scale linearity, enabling accurate phase decoding. Disadvantages are limited depth of field, which is less suitable for complex scenes, high power consumption, which is challenging for low-power devices, and high system cost, limiting its adoption in mass-market applications.

Before digital light modulation became mainstream, dynamic pattern generation relied heavily on mechanical scanning systems. Traditional galvanometric mirrors scanned laser beams in space to form patterns [260,261]. However, due to their bulky size and high cost, they were unsuitable for portable or embedded systems.

With the development of Micro-Electro-Mechanical Systems (MEMSs), micro-scanning mirrors have emerged as a compact, low-cost alternative. These MEMS devices are batch-fabricated using semiconductor-compatible processes (e.g., etching, electroplating), offering miniaturization, reliability, and low power consumption. While both DMDs and scanning mirrors are MEMS-based, the latter function as actuators for angular modulation, whereas DMDs operate more like LCD or LCoS arrays.

MEMS scanning mirrors were first used for 3D imaging in 2007 [262]. Intel released the first commercial MEMS-based product, RealSense SR300, in 2015 [263,264]. Revopoint followed in 2019 with a higher-precision MEMS 3D scanner [265]. Recent research by Li’s group includes error propagation modelling [266,267], high-resolution fringe projection via delayed superposition [268], attention-based end-to-end HDR structured light reconstruction [269], a window smoothing model for MEMS-based structured light reconstruction [266], anti-harmonic layered phase-shifting [245], and tailored calibration models for MEMS-based structured light systems [270,271,272].

As shown in Figure 16e, a typical single-axis MEMS scanner uses an edge-emitting laser (EEL) with collimation and beam-shaping optics (e.g., Powell or cylindrical lenses) to form a fan-shaped beam. A MEMS mirror then rapidly scans this beam angularly. When synchronized with laser modulation, this produces a 1D structured pattern on the target surface [263].

Two-axis MEMS mirrors can directly project 2D patterns with high flexibility [273]. In theory, well-designed MEMSs with quality laser sources can approximate the continuous fields of interferometric setups. Furthermore, laser illumination offers high linearity and signal-to-noise ratio for precise depth reconstruction. However, challenges such as speckle noise and scanning distortion persist, and current research aims to mitigate these through improved optics and control algorithms. Advantages of MEMS-based projection include low cost, which is suitable for consumer-scale deployment, being compact and highly integrated, which is ideal for embedded systems, high optical efficiency and energy utilization, a large depth of field which is adaptable to varied working distances, continuously tunable patterns which are ideal for phase-coded methods, and high frame rate and programmable projection. Its main limitation is that its effective spatial resolution is currently lower than that of DLP or interferometric systems due to speckle noise and mechanical distortions.

### 3.2. Micro- and Nano-Scale Surface Metrology

Micro- and nano-scale surface metrology employs diverse optical techniques for high-precision profiling of nanostructures. Interferometric microscopy (Twyman–Green, Michelson, Mach–Zehnder) enables direct surface shape and aberration measurements. Optical scatterometry extracts grating dimensions by analysing diffraction efficiency. Laser scanning confocal microscopy (LSCM) provides high axial resolution for complex freeform surfaces. Scanning white light interferometry (SWLI) reconstructs accurate 3D topography using low-coherence sources. Digital holographic microscopy (DHM) rapidly retrieves the phase and shape information of aspheric features. Near-field scanning, autofocus systems, and Fizeau inspection extend resolution, speed, and scalability for specialized applications.

**Figure 17:**
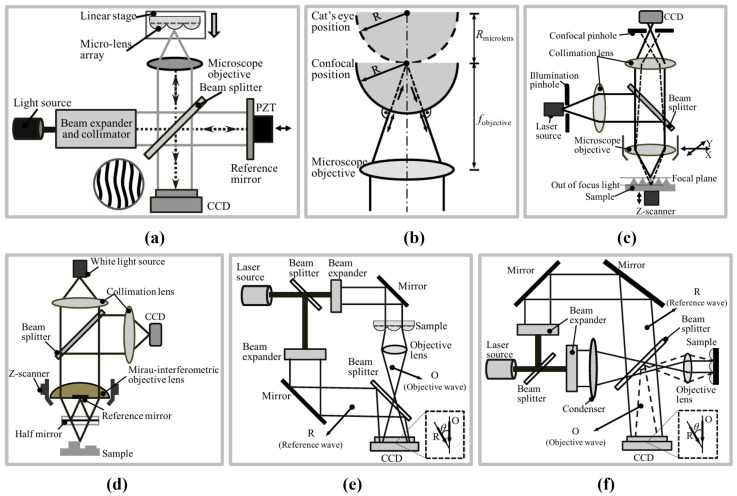
Schematic of major methods for nano-scale surface metrology. (**a**) Schematic of a phase-shifting TGI. (**b**) Measurement of the geometric properties of the micro-lens: radius of curvature, symmetric error, and shape deviation. (**c**) Schematic of a laser scanning confocal microscope. (**d**) Schematic of a scanning white light interferometer. (**e**) Experimental setups of DHM for transmission imaging. (**f**) Experimental setups of DHM for reflection imaging.

Optical interferometry plays a vital role in micro-optical surface characterization, with Twyman–Green, Michelson, and Mach–Zehnder interferometers being the most representative configurations [9,274]. Among them, the Twyman–Green interferometer is widely used for evaluating microlens arrays due to its high precision and controllability [275,276,277,278]. Figure 17a shows a typical phase-shifting Twyman–Green setup [276], where collimated beams are reflected off a reference mirror and the test sample, respectively. The resulting interference pattern is used to reconstruct the surface phase using phase-shifting techniques (e.g., π/2 phase steps driven by a PZT actuator). Deviations from the nominal shape yield geometric errors of the microlenses. The axial scanning of the sample enables the determination of curvature radius R. At the so-called cat’s eye position [Figure 17b], the incident beam is focused at the lens vertex and retro-reflected, producing straight fringes that minimize odd-order aberrations. Further translation to the confocal position allows for accurate focal length measurements.

Mach–Zehnder interferometers can sensitively detect wavefront distortion after transmission through the lens, revealing intrinsic aberrations [277]. Additionally, placing a mirror at the focal point of a lens in a Michelson setup allows for focal length uniformity evaluation, where fringe patterns captured by a CCD reveal the distribution of focal points across the array [275,279,280].

Optical scatterometry analyses microstructured surfaces by measuring light scattered under a controlled wavelength, polarization, and incident angle conditions. Key parameters such as the depth, linewidth, and duty cycle of gratings can be extracted [281,282,283]. Angular-resolved and integrating scatterometry are commonly used, and data are fitted against theoretical models to recover surface profiles [282].

Marciante et al. introduced the Symmetric Order Efficiency Ratio (SOER) method to infer groove geometry from the +1 and −1 diffraction orders [283]. Li et al. further proposed a duty cycle measurement technique that is independent of groove depth and requires no power measurement, making it suitable for subwavelength structures [284].

LSCM is a high-axial-resolution surface profiling technique based on point illumination and spatial filtering, suitable for imaging complex microstructures such as lenses, gratings, and freeform surfaces [285,286]. As illustrated in Figure 17c, a laser is focused onto the sample, and a pinhole in front of the CCD eliminates out-of-focus light. Lateral scanning is performed by an XY stage, while vertical scanning is achieved via a piezoelectric actuator. A full 3D surface can thus be reconstructed. For improved scanning stability and speed, DMD-based systems have been developed to enable multi-point parallel acquisition [287].

SWLI utilizes broadband, low-coherence light sources to perform interferometric surface measurements and is well suited for 3D profiling micro- and nano-scale features [279,288]. As shown in Figure 17d, a Mirau interferometer integrated into the objective splits the beam into reference and measurement paths. Interference fringes with maximal contrast appear when the optical path difference is minimized. Vertical scanning of the objective synchronizes with CCD imaging to reconstruct height information [289].

SWLI is ideal for characterizing the height of micro-lenses or step gratings [278,279]. However, challenges such as signal loss may arise at steep slopes or low-reflectivity regions [290].

DHM combines holographic imaging with numerical phase reconstruction, enabling fast, non-contact profiling of reflective or transmissive micro-optics [287,291,292,293]. Figure 17e,f shows typical setups for both transmission and reflection imaging [278]. The interference pattern formed by object and reference beams is recorded by a CCD, and the surface profile is reconstructed via phase-unwrapping algorithms. Surface properties such as diameter, modulation depth, and roughness can be extracted by fitting the data to a nominal spherical model. DHM is particularly advantageous for strongly aspheric or non-rotationally symmetric structures.

For large-area gratings, a rapid inspection method based on a Fizeau interferometer has been proposed [294,295]. The zeroth-order beam evaluates flatness, while first-order beams in X/Y directions are used to assess periodic deviations, enabling fast quality control of 2D diffractive optics.

Near-field Scanning Optical Microscopy (NSOM) overcomes the diffraction limit using a tapered, metal-coated fibre probe to operate in the optical near field [296,297,298,299]. The system comprises a piezo scanning stage and a tuning fork feedback system for height regulation. NSOM is well suited for phase imaging of biological and lithographic samples, particularly transparent or semi-transparent materials.

Additionally, autofocus imaging systems have been applied for freeform surface measurement [286]. These systems detect the defocus error in real-time and adjust the lens position along the optical axis to maintain optimal focus. Combined with XY stages and PZT feedback, 3D surface profiles can be rapidly acquired [300,301,302].

## 4. Conclusions and Outlook

### 4.1. Conclusions

Optical metrology has become an indispensable foundation for advanced manufacturing, where stringent demands for accuracy, efficiency, and reliability far exceed those of conventional production. Unlike traditional contact-based measurement methods that are limited by mechanical wear, slow speed, and potential surface damage, optical approaches provide non-contact, ultra-high precision, and rapid data acquisition capabilities. From the perspective of precision manufacturing, these advantages are not merely technical refinements, but fundamental enablers of next-generation industrial processes.

This review has organized optical metrology into two major application domains: precision positioning, and surface topography measurement. In precision positioning, laser interferometers, optical encoders, confocal methods, triangulation, and time-of-flight techniques offer scalable solutions for measuring linear and angular displacements, as well as multi-degree-of-freedom motions. These technologies directly support the sub-micron to nanometre-level positioning required by high-end equipment such as lithography stages, precision machine tools, and semiconductor assembly systems. In surface characterization, optical interferometry, structured light profilometry, and confocal microscopy represent mainstream approaches capable of bridging large-scale form evaluation and micro- and nano-scale surface inspection. Their ability to deliver accurate, rapid, and flexible measurements makes them particularly suitable for assessing freeform optics, semiconductor wafers, and functional micro-structured surfaces. By connecting these two domains, optical metrology provides a comprehensive measurement infrastructure that underpins both process stability and product performance in advanced manufacturing.

### 4.2. Outlook

Looking ahead, the evolution of optical metrology for precision manufacturing is expected to advance along several strategic directions.

First, cross-scale integration will be essential, enabling seamless transitions from macroscopic geometric inspection to micro- and nano-scale surface analysis on a single platform. Such capability will ensure that components ranging from metre-scale aerospace mirrors to sub-100 nm semiconductor features can be evaluated within a unified measurement framework.

Second, real-time and on-machine metrology will become increasingly critical. Embedding optical measurement modules directly into production equipment, such as CNC machines, additive manufacturing systems and semiconductor process lines, will support closed-loop control, where dynamic feedback optimizes process parameters instantaneously. This shift from offline inspection to online monitoring represents a paradigm change for manufacturing efficiency and quality assurance.

Third, hybrid and multimodal approaches are expected to overcome the inherent limitations of individual methods. By integrating interferometry, imaging, and spectral techniques, future systems will provide richer datasets, simultaneously capturing geometry, surface roughness, and material composition. Such comprehensive measurements will be particularly valuable for complex, multifunctional components.

Fourth, the integration of artificial intelligence will significantly enhance the intelligence and autonomy of metrology systems. AI-driven algorithms can adaptively adjust measurement strategies in response to environmental disturbances or surface complexity, while also accelerating data processing for large-scale, high-throughput applications.

Finally, miniaturization and on-chip integration of optical measurement modules will promote their adoption in portable devices and embedded applications. Following trends in MEMS and photonic integration, compact optical metrology solutions will improve accessibility, reduce cost, and expand applicability to decentralized or resource-limited manufacturing environments.

In summary, this review has approached optical metrology from the perspective of precision manufacturing, not only emphasizing technical principles, but also application-driven requirements. The continuing development of cross-scale, real-time, and intelligent metrology will reinforce its role as a cornerstone technology, enabling the transition toward smarter, faster, and more precise manufacturing systems.

## Figures and Tables

**Table 1:** Comparison of optical measurement methods for linear displacement in advanced manufacturing.

Method	Typical Accuracy	Typical Application Scenarios	Advantages and Disadvantages
Laser Interferometer	Nanometre to picometre level; measurement range from tens of meters to several kilometre	Large-scale ultra-high-precision linear positioning (lithography stage, synchrotron beamline, calibration of precision machine tool axes)	Theoretical accuracy is extremely high, as it is fundamentally based on the optical wavelength. Very large measurement range. Multi-wavelength or optical frequency comb techniques can achieve absolute measurement. Highly sensitive to environmental conditions, since the air refractive index is strongly affected by temperature and humidity. Multi-axis measurements require multiple subsystems, resulting in low industrial integration and high cost.
Optical Linear Encoder	Incremental interferometric type: picometre level (e.g., 6.1 pm); absolute type: ∼1 nm; measurement length typically ≤20 m (up to 40 m in special designs)	Position feedback for machine tools, semiconductor lithography stages, coordinate measuring machines (CMMs), automated production line positioning	Compact structure and easy integration; multi-degree-of-freedom measurements can be implemented within a single subsystem. Stable performance and insensitivity to environmental changes. Absolute type retains position data after power-off. Measurement range limited by grating size. High-precision, long-travel designs are costly to manufacture.
Confocal Measurement	Nanometer-level resolution in commercial systems (e.g., 12 nm); measuring range typically ≤ tens of millimeters	Thickness measurement of transparent multilayer structures, deep-hole inspection, microstructure surface measurement, MEMS inspection, height measurement in precision assembly	High axial resolution. Suitable for highly reflective or transparent materials. Compact structure, capable of high-speed measurement. Measures only axial displacement. Signals easily lost on highly tilted or low-reflectivity surfaces. Limited measuring range.
Laser Triangulation	Micrometre to sub-micrometre level (depending on sensor resolution and optical design)	Industrial profile scanning, surface topography inspection, position feedback, large-stroke in-line displacement measurement	Long working distance and flexible installation. Strong adaptability to target surfaces (capable of measuring diffusive or specular reflection). Low cost and high measurement speed. Accuracy limited by CCD/CMOS resolution. Surface roughness, texture variations, and illumination changes can introduce speckle noise. Accuracy decreases over long travel ranges.
Time-of-Flight (TOF) Measurement	Conventional TOF: ∼100 μm; femtosecond pulse interferometry/BOC: nanometre to sub-nanometre level	Large-scale distance measurement (conventional TOF), medium- to long-range high-precision positioning (femtosecond-laser dual-comb), satellite formation navigation	Absolute distance measurement directly based on the definition of the speed of light. Femtosecond/dual-comb techniques enable high precision with long measurement range. Suitable for dynamic measurements. Conventional TOF has limited accuracy. Femtosecond/dual-comb systems are costly and structurally complex, requiring extremely high system stability.

**Table 2:** Comparison of optical measurement methods for angular displacement in advanced manufacturing.

Method	Typical Accuracy	Typical Application Scenarios	Advantages and Disadvantages
Rotary Encoder	∼$10^{-5}$ rad	Rotary axis positioning of machine tools, angular feedback for rotary tables, joint angle detection in robots	Mature commercial products, compact structure, high resolution, low cost, easy integration with control systems. Limited to fixed rotation axes, installation position constraint, sensitive to contamination and vibration.
Autocollimator	∼$10^{-6}$ rad	Flatness adjustment, angular deviation inspection, optical platform attitude adjustment, rotary accuracy check, multi-degree-of-freedom tilt measurement	Non-contact, high resolution, capable of measuring tilting without a fixed rotation axis, widely used in multi-DOF measurement. Requires sufficient surface reflectivity and stable optical path, sensitive to temperature and vibration.
Interferometric Methods	∼$10^{-9}$ rad	Multi-DOF measurement of precision stages, roll angle detection in nanometer positioning systems, high-accuracy tilt measurement	Extremely high resolution, capable of measuring displacement and angle simultaneously, suitable for ultra-precision applications, supports absolute/incremental modes. Complex optical setup, high alignment requirements, limited range for some methods, high environmental control demand.

## Data Availability

The data presented in this study are available on request from the corresponding author.
